# Fentanyl Structure as a Scaffold for Opioid/Non-Opioid Multitarget Analgesics

**DOI:** 10.3390/ijms23052766

**Published:** 2022-03-02

**Authors:** Piotr F. J. Lipiński, Joanna Matalińska

**Affiliations:** Department of Neuropeptides, Mossakowski Medical Research Institute Polish Academy of Sciences, Pawińskiego 5, 02-106 Warsaw, Poland; jmatalinska@imdik.pan.pl

**Keywords:** fentanyl, heterodimers, multitarget drugs, pain, structure-activity relationships

## Abstract

One of the strategies in the search for safe and effective analgesic drugs is the design of multitarget analgesics. Such compounds are intended to have high affinity and activity at more than one molecular target involved in pain modulation. In the present contribution we summarize the attempts in which fentanyl or its substructures were used as a μ-opioid receptor pharmacophoric fragment and a scaffold to which fragments related to non-opioid receptors were attached. The non-opioid ‘second’ targets included proteins as diverse as imidazoline I_2_ binding sites, CB_1_ cannabinoid receptor, NK_1_ tachykinin receptor, D_2_ dopamine receptor, cyclooxygenases, fatty acid amide hydrolase and monoacylglycerol lipase and σ_1_ receptor. Reviewing the individual attempts, we outline the chemistry, the obtained pharmacological properties and structure-activity relationships. Finally, we discuss the possible directions for future work.

## 1. Introduction

Finding novel drugs for effective and safe management of severe and/or chronic pain poses a major challenge for modern medicinal chemistry and pharmacology. The key element of our current therapeutical toolbox against pain are agonists of the μ-opioid receptor (MOR). These, while being highly effective in severe acute conditions, are not devoid of adverse effects that turn out most problematic with prolonged use. Many patients taking opioids suffer from sedation, nausea, hard-to-treat constipations, paradoxical hyperalgesia or endocrinologic dysfunctions [1,2]. Long-term opioid use increases the risk of developing physical dependence and addiction [1]. Tolerance to opioid analgesia (but not to the opioid side effects) appears relatively quickly [3], requiring escalation of the dosage, but this in turn exacerbates the mentioned side effects. Moreover, the use of classical opioids in neuropathic pain conditions is often of limited effectiveness [4].

**Figure 1:**
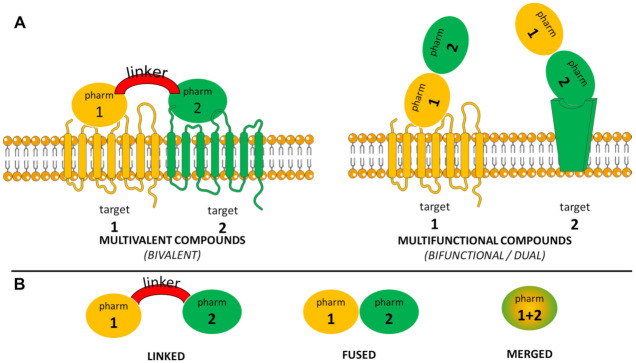
(**A**) Schematic distinction between multivalent and multifunctional compounds. (**B**) Schematic representation of various types of multitarget compounds according to the degree to which the included pharmacophores are integrated. ‘Pharm’ stands for ‘pharmacophore’.

Several strategies have been devised with the hope of achieving effective opioid analgesia with improved side effects profile [5]. One that over the years has enjoyed a good deal of interest from the researchers is the development of multitarget analgesic (MTA) compounds [6,7]. Substances of this type have significant affinity and activity at more than one molecular target involved in pain modulation. Among MTAs one can distinguish multifunctional and multivalent compounds (Figure 1A) [7]. Multivalent (usually bivalent) compounds are able to bind to a few molecular targets at the very same time, for example by targeting heterodimers formed by opioid receptors with other receptors [8]. On the contrary, multifunctional (usually bifunctional, dual) compounds possess high affinity to more than one target but bind to each of these in separate.

That a multitarget pharmacological profile could be therapeutically beneficial for analgesics arises from the complex nature of many pain conditions which involves interplay between numerous signalling pathways. The interplay between pro- and antinociceptive factors is also thought to be responsible for analgesic tolerance, hyperalgesia, and low efficacy of opioids in neuropathic pain [9]. By simultaneous targeting of MOR and an additional receptor, a multifunctional analgesic could counteract the side effects directly or indirectly, in the latter case by improving efficacy and thus lowering the need for the activation of opioid pathways. Additional signalling components are also sometimes expected to provide satisfactory activity in neuropathic pain.

In the search for MTAs, compounds targeting many diverse pairs of molecular targets have been obtained [6]. The usual ‘major’ target in these pairs is the μ-opioid receptor (MOR). The auxiliary targets may be other G-protein coupled receptors (GPCRs), e.g., CB_1_ cannabinoid receptor [10,11], NK_1_ tachykinin receptor [12,13], D_2_ dopamine receptor, CCK_2_ cholecystokinin receptor [14,15], neurotensin receptor [16,17], MC_4_ melanocortin receptor [18,19], neuropeptide FF receptor [20] or α_2_-adrenergic receptor [21]. The second target could be also a non-GPCR receptor (σ_1_ receptors [22]), an enzyme (cyclooxygenases [23], fatty acid amide hydrolase and monoacylglycerol lipase [24]), an ion channel (voltage gated calcium channels [25]) or a binding site of a less clear character (imidazoline I_2_ binding sites [26,27,28]). Moreover, a separate and a well-developed subfield are MTAs aimed at targeting of two or more different opioid receptor subtypes [7].

MTAs (or multitarget drugs in general) are designed by combining pharmacophores of the two (or more) desired molecular targets. Depending on the degree to which the structural elements of both pharmacophores are integrated, one can speak of ‘linked’, ‘fused’ or ‘merged’ dual ligands (Figure 1B) [29,30]. In the ’linked’ ligands, the structural fragments related to the individual targets are joined by a linker/spacer (sometimes a very long one). In the ’fused’ molecules, the fragments are directly combined, and no spacer can be discerned. In the ‘merged’ ligands, there is an at least partial overlap of the pharmacophoric elements.

**Figure 2:**
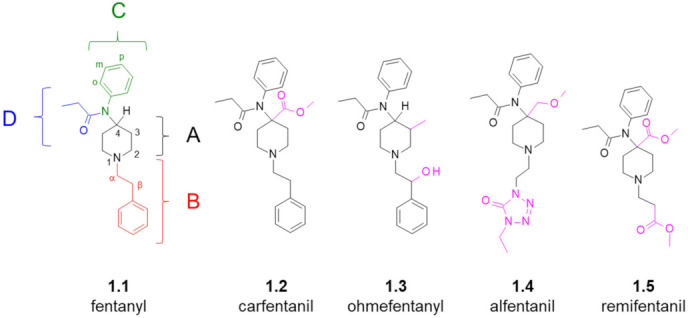
Structure of fentanyl (with its logical dissection and position numbering convention shown) and of a few important analogues thereof. Modifications (compared to the parent compound) are marked in pink.

Whichever approach one is going to follow in their search for MTAs, a key issue is the choice of the pharmacophoric fragments to be used. In the present contribution, we shall summarize the attempts to create MTAs that (directly or indirectly) utilized fentanyl (**1.1**, Figure 2) or its substructures as a μ-opioid pharmacophore and a scaffold to append (or to melt into) pharmacophoric elements of other, non-opioid molecular targets. 

Fentanyl (*N*-phenyl-*N*-[1-(2-phenylethyl)piperidin-4-yl]propanamide, **1.1**) is a very useful and a well-established analgesic and anaesthetic drug [31]. The compound has high affinity for MOR and displays potent agonistic properties at this receptor. It is also very lipophilic and thanks to this it readily distributes into the central nervous system (CNS), rapidly producing the opioid effect. Depending on the particular testing conditions, fentanyl may be 50 to 100 times more potent an analgesic than morphine [32]. In clinical settings (in low doses, with short-term use), fentanyl is rather safe, although illicit recreational use is associated with thousands of ‘fentanyl deaths’ each year [33]. 

**Scheme 1:**
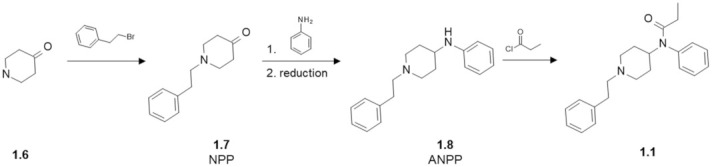
Exemplary synthetic route to fentanyl. Many basic analogues can be accessed by modifications of the alkylating agent, amine or acylating agent. **NPP**—*N*-phenethylpiperidin-4-one. **ANPP**—4-anilino *N*-phenethyl-piperidine.

The synthesis of fentanyl and of many basic analogues can be conveniently accomplished in three steps (Scheme 1) as demonstrated by an optimized method of Valdez et al. [34]. In Step I, piperidin-4-one (**1.6**) is *N*-alkylated with e.g., 2-phenethyl bromide. In Step II, the resulting *N*-phenethylpiperidin-4-one (**NPP**, **1.7**) is subject to reductive amination with aniline to yield (via a Schiff base) 4-anilino *N*-phenethyl-piperidine (**ANPP**, **1.8**). Finally, the amine **1.8** is acylated using e.g., propionyl chloride. Alterations of the alkylating agents, amines or the acylating agents provide access to many fentanyl analogues, while leaving this basic synthetic scheme untouched. The original fentanyl syntheses [35,36] as well as syntheses found in many other papers for other analogues tend to utilize *N*-protected piperidin-4-ones, which are deprotected and *N*-alkylated only after other desired elements have been introduced. 

In terms of structure (Figure 2), the core of fentanyl is the piperidine ring (region **A**). In position 1, this ring is decorated with the phenethyl group (region **B**), while attached in position 4 is a nitrogen atom substituted with a phenyl ring (region **C**) and a propionyl group (region **D**). Over the years, this elementary structure has been thoroughly explored and numerous analogues of fentanyl (“fentanyls” or “fentalogues”) were synthesized for probing SAR of the 4-anilidopiperidine class of analgesics. A recent concise SAR and chemistry summary was provided by Vardanyan and Hruby [37]. Here, let us mention only that the following modifications/substitutions could be found in the most potent derivatives:

(region **A**): 4-carboxymethyl, 4-methoxymethyl, 3-methyl,
(region **B**): α-methyl, β-hydroxyl (if accompanied by 3-methyl in the region **A**), replacement of the phenyl ring for heterocyclic aromatics,
(region **C**): *p*-fluoro substitution at the ring (some other substitutions and replacements could be tolerated or beneficial, too).
(region **D**): alicyclic fragments (e.g., cyclopropyl), linear (elongated) or branched alkyl chains, ether fragments, aromatic fragments (e.g., 2-furanyl).
Particularly worth pinpointing seem such interesting analogues as ultrapotent μOR agonists, such as carfentanil (**1.2**, [38]) or ohmefentanil (**1.3**, [39]), and ultrashort acting analgesics, such as alfentanil (**1.4**, [40]) or remifentanil (**1.5**, [41]).

That the structure of fentanyl (**1.1**) may be a good starting point for creating MTAs derives from (1) its pharmacological properties, (2) a relatively facile chemistry by which diverse analogues and functionalized derivatives can be accessed, (3) wealth of available structure-activity relationships (SAR) data.

## 2. Fentanyl-Based MTAs Targeting MOR and I_2_-Imidazoline Binding Sites

Historically, the first attempt to utilize the fentanyl scaffold for creating multitarget opioid/non-opioid compounds was the one in which researchers tried to obtain dual ligands for MOR and I_2_-imidazoline binding sites (I_2_-IBS) [26,27,28].

**Figure 3:**
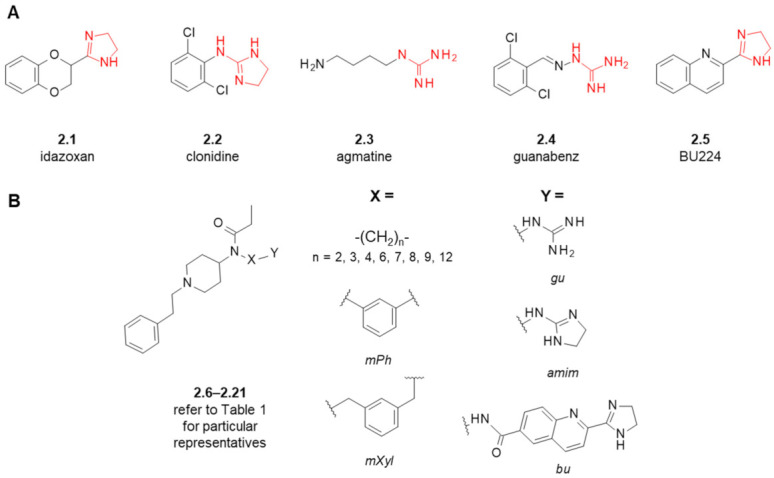
(**A**) Examples of typical I_2_-IBS ligands. In red marked are 2-aminoimidazoline, imidazoline and guanidine fragments characteristic for these compounds. (**B**) General scheme of fentanyl-based MOR/I_2_-IBS ligands based on the fentanyl scaffold. Refer to Table 1 for particular representatives.

Both the nature and the role of I_2_-IBS has remained elusive. According to Regunathan and Reis [42], I_2_ imidazoline binding sites (receptors) are nonadrenergic binding sites that have high affinity for [^3^H]-idazoxan (**2.1**, Figure 3A) and a substantially lower affinity for [^3^H]-clonidine (**2.2**) or [^3^H]-*para*-aminoclonidine. Rather than being a single protein, I_2_-IBS seem to represent a heterogenous population of binding sites [43]. Their identity is still not conclusively established. In 2009, a brain creatine kinase (B-CK) was found to be an I_2_ imidazoline binding protein [44], but several other I_2_-binding sites were immunodetected and some are suspected to be allosteric binding sites on monoamine oxidases A and B [43]. 

From the standpoint of pharmacology, I_2_-IBS ligands are considered for their neuroprotective actions and for their antinociceptive effects in some models of chronic and neuropathic pain [45,46]. So far, no approved drug has been developed based on imidazoline receptor concept, but an I_2_-IBS agonist CR4056 has some chance of becoming one, since recently it has successfully passed the Phase 2 clinical trial for chronic pain associated with osteoarthritis [47]. 

Apart from their analgesic action as single agents, I_2_-imidazoline agonists may be adjuvants to opioids. Simultaneous administration of both had been shown to produce synergistic antinociceptive effect and to attenuate tolerance to opioid action [45,46]. Based on this, a Spanish group proposed that development of hybrid molecules binding to both MOR and I_2_-IBS might be an interesting strategy for finding novel analgesic compounds with improved properties [26,27,28]. They presented fentanyl derivatives designed to have affinity for both these targets. 

Since some typical imidazoline receptor ligands (e.g., clonidine (**2.2**), agmatine (**2.3**), guanabenz (**2.4**), Figure 3A) contain guanidino or 2-aminoimidazolino groups in their structures, an attempt to achieve I_2_ affinity was performed by introducing such groups into the fentanyl structure (Figure 3B and Table 1). First, these groups were either mounted on *meta*-position of the aromatic ring in region C (**2.6** and **2.7**) or connected to the amide nitrogen via spacer made of three -CH_2_- units (**2.8** and **2.9**) [26]. Following the initial activity data, the authors further explored SAR of the guanidine series by varying the length and the nature of the spacer (**2.10**–**2.17**) [27,28]. Finally, an I_2_-IBS selective ligand, BU224 (**2.5**) was coupled to the principal scaffold by aliphatic linkers of a variable length (**2.18**–**2.21**) [28].

**Scheme 2:**
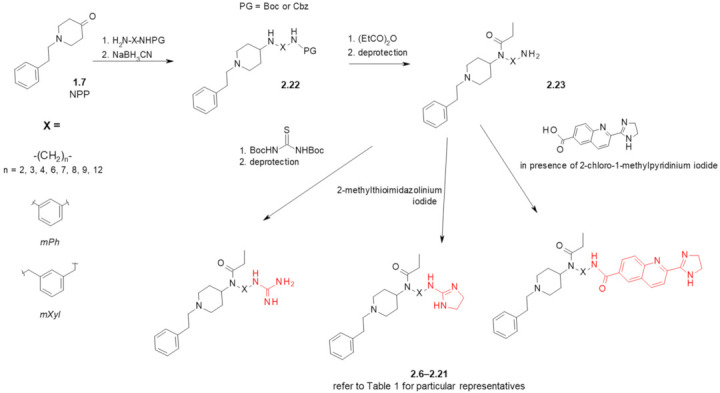
Preparation of fentanyl-based MOR/I_2_-IBS ligands. See Figure 3 and Table 1 for particular representatives. In red marked are fragments characteristic for I_2_-IBS ligands. Boc—*tert*-butyloxycarbonyl, Cbz—carboxybenzyl.

The reported routes to the designed hybrids (Scheme 2 and Scheme S1 in Supplementary Materials) started with *N*-phenethyl-4-piperidinone (**NPP**, **1.7**). **NPP** was subject to reductive amination with mono-protected diamines (or with 3-nitroaniline on the route to **2.6** and **2.7**, Scheme S1). The resulting aminopiperidines (**2.22**) were acylated with propionic anhydride and deprotected with trifluoroacetic acid (for Boc-protected derivatives) or by catalytic hydrogenation (for Cbz-protected derivatives). The latter reaction served to reduce 3-nitro group to 3-amino group on the route to **2.6** and **2.7**, too. Compounds **2.23** with a free amino group on an aliphatic or aromatic pendant were then (a) guanidinated with *N*,*N*′-di(*tert*-butyloxycarbonyl)thiourea and deprotected, (b) treated with 2-methylthioimidazolinium iodide or (c) coupled with an acid derivative of BU224 in the presence of Mukaiyama’s reagent (2-chloro-1-methylpyridinium iodide).

**Table 1:** Affinities of fentanyl-based MOR/I_2_-IBS ligands and reference compounds for the intended molecular targets.

	Structure (Refer to Figure 3B for Structural Explanations)			Affinity [K_i_ (nM)] ^1^		
Compound	X	Y	d_NC_^2^	MOR ^3^	I_2_-IBS ^4^	Ref.
**fentanyl (1.1)**	-	-	-	6 ± 1.5 ^5^	5462 ± 1343 ^6^	[26]
				2.9 ± 1.5	8593 ± 738	[28]
**idazoxan (2.1)**	-	-	-	-	307 ± 183 ^6^	[26]
					28 ± 11	[27,28]
**BU224 (2.5)**	-	-	-	-	9.8 ± 0.3	[48]
**2.6**	mPh	gu	5	7.8 ± 2.5 ^5^	1890 ± 499 ^6^	[26]
**2.7**	mPh	amim	5	7119 ± 4089 ^5^	9630 ± 6731 ^6^	[26]
**2.8**	-(CH_2_)_3_-	gu	5	37 ± 12 ^5^	2022 ± 949 ^6^	[26]
				23 ± 4.5	1920 ± 996	[27]
**2.9**	-(CH_2_)_3_-	amim	5	1751 ± 1135 ^5^	2327 ± 811 ^6^	[26]
**2.10**	-(CH_2_)_2_-	gu	4	433 ± 83	437 ± 228	[27]
**2.11**	-(CH_2_)_4_-	gu	6	0.59 ± 0.18	>10,000	[28]
**2.12**	-(CH_2_)_6_-	gu	8	1.04 ± 0.28	409 ± 238	[27]
**2.13**	-(CH_2_)_7_-	gu	9	0.37 ± 0.19	6627 ± 3106	[28]
**2.14**	-(CH_2_)_8_-	gu	10	37 ± 9.7	126 ± 72	[27]
**2.15**	-(CH_2_)_9_-	gu	11	26 ± 6	58 ± 46	[28]
**2.16**	-(CH_2_)_12_-	gu	14	477 ± 75	6.5 ± 3.0	[27]
**2.17**	mXyl	gu	7	0.0098 ± 0.0033	>10,000	[28]
				0.448 ± 0.079 ^7^		[49]
**2.18**	-(CH_2_)_3_-	bu	12	6142 ± 2123	875 ± 713	[28]
**2.19**	-(CH_2_)_6_-	bu	15	2168 ± 66	323 ± 270	[28]
**2.20**	-(CH_2_)_8_-	bu	17	339 ± 35	>10,000	[28]
**2.21**	-(CH_2_)_12_-	bu	21	545 ± 179	547 ± 316	[28]

^1^ K_i_, inhibition constant (nM) with standard error of the mean, ^2^ d_NC_—topological distance (number of bonds) between the nitrogen attached at the position 4 of the piperidine ring and the central carbon atom in guanidine, 2-aminoimidazoline or imidazoline moieties, see Figure S1C, ^3^ unless specified otherwise, competitive assays done in membrane preparations of post-mortem human frontal cortex, 2 nM [^3^H]DAMGO as radioligand, ^4^ unless specified otherwise, competitive assays done in membrane preparations of post-mortem human frontal cortex, 1 nM [^3^H]2-BFI as radioligand, ^5^ competitive assays done in neural membrane preparations of mice brain, 2 nM [^3^H]DAMGO as radioligand, ^6^ competitive assays done in neural membrane preparations of mice brain, 1 nM [^3^H]2-BFI as radioligand, ^7^ competitive assays done in membrane preparations of rat brain, 0.72 nM [^3^H]DAMGO as radioligand.

The analogues with the guanidine moiety exhibited diversified MOR affinities with K_i_’s ranging from subnanomolar to single digit micromolar ones (Table 1). Guanidine derivatives with medium-length spacers (6–9 bonds) were of affinity similar or better than that of fentanyl (**1.1**). Shorter or longer spacers gave a monotonic decrease in binding affinity for guanidine-bearing analogues (Figure S1A in Supplementary Materials). Both considered 2-aminoimidazoline derivatives (**2.7** and **2.9**) had a MOR K_i_ greater than 2 μM. In the cases of BU224 hybrids, the MOR K_i_ varied between 339 and 6142 nM. The most potent MOR binder of the whole set was the guanidine derivative based on *meta*-xylene bridge (**2.17**) for which the authors reported a picomolar K_i_ [28]. Later, this analogue (**2.17**) was retested by Weltrowska et al. [49] who found somewhat lower, but still subnanomolar MOR affinity (K_i_ = 0.448 nM) [49]. Interestingly, compound **2.17** was found to possess an equally good binding to kappa opioid receptor (KOR, K_i_ = 0.536 nM) [49]. 

According to our correlational analysis of the reported MOR affinities (Figure S1), there is a bilinear (or reversed U-shaped) dependence of the affinity on the linker length (particularly clearly seen for the guanidine series, Figure S1A). This suggests that in the MOR binding site there is a good interaction partner for guanidine/imidazoline that can be reached by the analogues in which these functions are attached at a linker of appropriate length. According to modelling by Weltrowska et al., such an interaction partner could be Asp216 side chain located at the second extracellular loop of MOR [49].

Regarding the affinity for I_2_-IBS, for fentanyl itself (**1.1**), K_i_ values of 5462 and 8593 nM were found [26,28]. These values are significantly worse than the inhibition constants found for a reference I_2_-ligands like, idazoxan (**2.1**, K_i_ = 307 nM [26] or K_i_ = 28 nM [28]) or BU224 (**2.5**, K_i_ = 9.8 nM [28]). Most of the fentanyl-hybrids had moderate to very low affinities. For only two of them (guanidine derivatives with nine or twelve methylene units in the linker, **2.15** and **2.16**) the inhibition constants reported were below 100 nM. The analogues with 2-aminoimidazoline moiety (**2.7** and **2.9**) had low affinity (K_i_ > 1 μM). All four BU224-based hybrids suffered a significant decrease in I_2_-IBS binding compared to their prototype, with K_i_’s from 323 nM to greater than 10,000 nM. Notably, a subnanomolar MOR binder **2.17** was reported to have K_i_ > 10,000 nM at I_2_-IBS. If any SAR trend could be found in these data, this would be that for guanidine derivatives the I_2_-IBS affinity is positively correlated with the linker length (Figure S1B). Thus, the trend in I_2_-IBS affinity is not parallel to the putative trend for MOR affinities (Figure S1D) and optimizing the affinity ratios could not be expected with simple modulation of the linker lengths.

As to functional activity, analogues **2.6** and **2.8** assayed in isolated tissues (inhibition of electrically induced contractions in longitudinal muscle/myenteric plexus, LM/MP, from guinea pig ileum, GPI) turned out to be MOR agonists, however weaker than morphine (EC_50_ values: 1.9 μM, 6.61 μM and 0.21 μM for **2.6**, **2.8** and morphine, respectively) [26]. Two compounds with high MOR affinity and tolerable I_2_-IBS affinity (**2.12** and **2.21**) were evaluated in [^35^S]GTPγS functional assays on membranes of post mortem human frontal cortex. The guanidine derivative **2.12** turned out to be a MOR agonist of rather low potency (25% stimulation of [^35^S]GTPγS binding; reverted by naloxone; EC_50_ = 4.21 μM compared to DAMGO EC_50_ = 77.1 μM). In the case of BU224-based analogue **2.21** much higher stimulation was observed (+ 125%), but the effect was not sensitive to the presence of naloxone, whence it can be concluded that this activity was MOR-independent.

The analogues **2.6**, **2.8** and **2.12** were tested further for analgesic activity in hot plate and writhing test in mice after the intraperitoneal administration (ip) [26,28]. The former two were relatively active in the writhing test (but less active than morphine), while inactive in the hot plate test (in nontoxic doses). Despite decent MOR affinity, compound **2.12** displayed no analgesic effect up to 40 mg/kg in either test and this high dosage turned out to be significantly lethal. The authors noted that this could be explained either based on rather low efficacy shown by **2.12** in the functional test or since a dicationic compound might have poor blood-brain barrier penetration.

## 3. Fentanyl-Based MTAs Targeting MOR and CB_1_R

The above-described research on MOR/I_2_-IBS ligands, apart from its important exploratory and pioneering character, produced SAR data and chemistry potentially useful for other attempts of ‘multitargeting’ with the fentanyl scaffold. The same Spanish group who generated these data used it to create molecules able to bind with MOR and cannabinoid 1 receptor (CB_1_R) [50].

Just as MOR, CB_1_R is a GPCR widely expressed both in the CNS and in the periphery. Both receptors are involved in the control of nociception, mood, behaviour and food intake. There is much evidence on possible bidirectional interplay between cannabinoid receptors and MOR (nicely summarized in a review by Zádor and Wollemann [51]). The proteins are expressed in the same CNS areas, and they can be localized at the same neurons. In vitro, MOR/CB_1_R heterodimers are formed. Some CB_1_R antagonists reverse the morphine-induced analgesia, while antinociception produced with tetrahydrocannabinol (a CB_1_R/CB_2_R ligand) can be blocked with opioid antagonist naloxone. Importantly, development of tolerance to morphine may be inhibited by some CB_1_R antagonists [52]. These facts prompted the development of MOR/CBR hybrid ligands made of peptide or alkaloid opioid fragments linked to CB_1_R or CB_1_R/CB_2_R pharmacophores [10,11,53]. 

**Figure 4:**
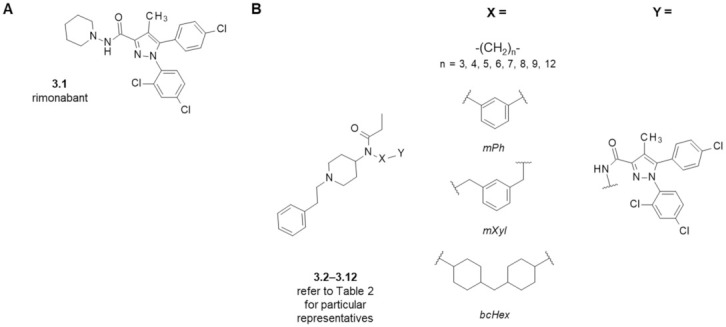
(**A**) Structure of a selective CB_1_R ligand, rimonabant. (**B**) General scheme of fentanyl-based MOR/CB_1_R ligands based on the fentanyl scaffold. Refer to Table 2 for particular representatives.

**Scheme 3:**
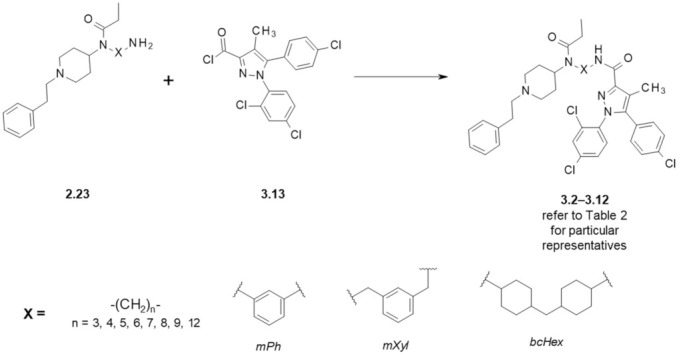
Preparation of fentanyl-based MOR/CB_1_R ligands. See Table 2 for particular representatives.

The attempt with the fentanyl scaffold [50] was meant to attach a CB_1_R pharmacophore by similar diamine linkers that previously had served to obtain compounds **2.6**–**2.21**. The CB_1_R fragment was based on rimonabant (**3.1**, Figure 4A), a selective CB_1_R inverse agonist/antagonist (once in clinical use but withdrawn). SAR studies of **3.1** suggested that replacement of the piperidine ring by alkyl chain was tolerated by CB_1_R. Hence, derivatives **3.2**–**3.12** were designed. Their synthesis (Scheme 3) utilized the intermediates (**2.23**) whose preparation was described earlier in the works on MOR/I_2_-IBS ligands (Scheme 2) [26,27,28]. To obtain **3.2**–**3.12** (Scheme 3), the free amino group of the appropriate analogues **2.23** was acylated by an acid chloride derivative of the rimonabant core (**3.13**).

Binding affinity assays (Table 2) revealed that all hybrids **3.2**–**3.12** had diminished CB_1_R affinity compared to rimonabant (**3.1**). Submicromolar K_i_’s at cannabinoid 1 receptor were found for propyl (**3.2**), butyl (**3.3**) and heptyl-based (**3.6**) compounds, whereas the longest derivatives (**3.8** and **3.9**) or those with aromatic spacers (**3.10** and **3.11**) did not appreciably bind to the receptor. For the alkyl derivatives, an approximate, linear, negative relationship between the linker chain length and CB_1_R affinity can be proposed (Figure S2B).

As to the MOR affinity, the hybrids were significantly worse binders than the parent fentanyl (**1.1**). or the corresponding guanidine derivatives (**2.6**–**2.16**) from MOR/I_2_-IBS works [26,27,28]. The K_i_ ranged from ~100 nM to ~7 μM. Submicromolar values were found for **3.4**, **3.5**, **3.8** and **3.12**. If the pK_i_’s are plotted against the chain length, a zig-zag pattern with two optima could be supposed (Figure S2A). This would suggest the existence of two separate subsites in which rimonabant fragment could enjoy relatively favourable interactions with the MOR (Figure S2C). Again, as in the MOR/I_2_-IBS hybrids, the affinity trends for CB_1_R and MOR are not parallel (Figure S2D).

The compounds **3.3**, **3.5** and **3.6** were advanced to functional assays ([^35^S]GTPγS binding). Consistently with the design assumption, they were found to be CB_1_R antagonists of potency similar to that of rimonabant [50]. Quite surprisingly however, these analogues turned out to be opioid antagonists. For **3.5** and **3.6**, tentative behavioural in vivo tests confirmed the CB_1_R and MOR antagonistic properties. In mice, the compounds **3.5** (4 mg/kg ip) and **3.6** (5 mg/kg ip) were able to antagonise the effects that WIN55,212-2 (a potent cannabinoid agonist; at dose 1.5 mg/kg) had on rectal temperature, catalepsy, pain perception and spontaneous activity. Similarly, they blocked morphine analgesia in a hot plate test (10 mg/kg ip prior to 10 mg/kg morphine, ip). Since both CB_1_R and MOR antagonism is known to influence alcohol dependence [57], the authors checked if **3.6** could affect ethanol self-administration (alcohol relapse model in Wistar rats), but no significant effect was observed up to 8.0 mg/kg.

The MOR antagonism confirmed for a few analogues seems particularly worth noting since opioid antagonism in fentanyl-based compounds is rather uncommon. In the numerous family of fentanyls, only few such examples are known [58,59]. This is in marked contrast to the alkaloid opioid receptor ligands, among which many compounds with varying functional properties have been described.

## 4. Fentanyl-Based MTAs Targeting MOR and NK_1_R

A GPCR which has been many times used as a second target for MTAs is the NK_1_ tachykinin receptor (NK_1_R). An endogenous agonist of this receptor, Substance P (SP), is a sensory neurotransmitter involved in the pain perception, usually considered to be a pronociceptive factor [60]. Upregulation of SP and NK_1_R after prolonged opioid intake as well as in the chronic pain conditions is believed to be involved in the development of central sensitization, hyperalgesia and opioid analgesic tolerance [61,62]. Simultaneous administration of NK_1_R antagonists with opioid agonists was reported to give improved antinociceptive response and to prevent antinociceptive tolerance [63]. On the other hand, there are some data which indicate that in certain conditions SP, its metabolites or selective NK_1_R agonists could have analgesic activity, too [64]. 

Hence, many opioid/NK_1_R multifunctional ligands were prepared and tested. These contained both peptide and organic structural fragments and were intended to exhibit either agonistic or antagonistic properties at the NK_1_R. A review of the NK_1_R-related multifunctional analgesics and a critical evaluation of the concept was provided recently by Kleczkowska et al. [12]. 

**Figure 5:**
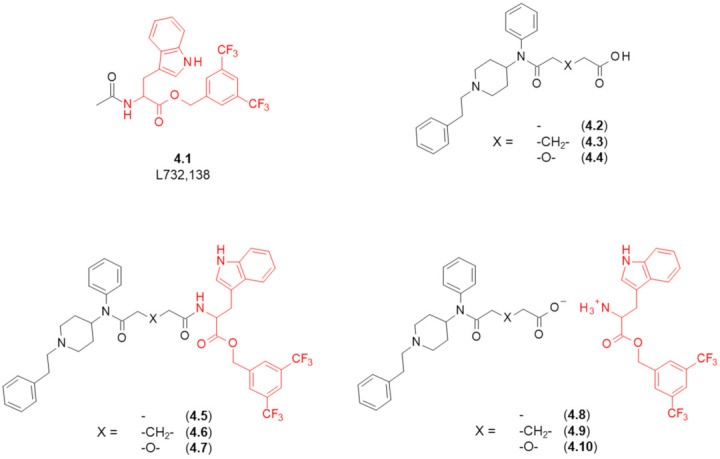
Structures of NK_1_R antagonist L732,138, carboxylate derivatives of fentanyl and of the designed fentanyl-based MOR/NK_1_R ligands. In red marked are fragments responsible for NK_1_R binding.

**Scheme 4:**
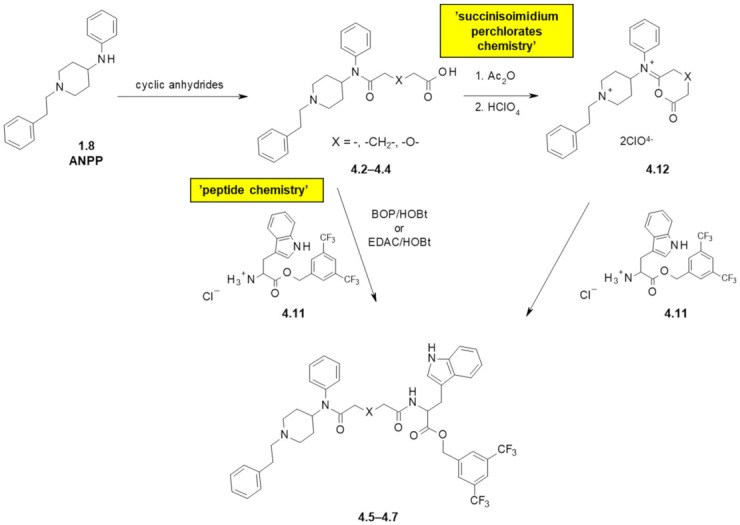
Preparation of fentanyl-based MOR/NK_1_R ligands. See Figure 5 and Table 3 for particular representatives. BOP—benzotriazole-1-yl-oxytris(dimethylamino)-phosphonium hexafluorophosphate, HOBt—1-hydroxybenzotriazole, EDAC—1-ethyl-3-(3-dimethylaminopropyl)carbodiimide.

Vardanyan et al. examined whether dual MOR/NK_1_R ligands could be created using the fentanyl scaffold [65]. The NK_1_R pharmacophoric fragment to be employed was based on the structure of one of the early potent NK_1_R antagonists, L732,138 (**4.1**, Figure 5, [66]). The authors chose carboxyfentanyl (**4.2**) and its two analogues (**4.3**–**4.4**) to serve for attaching the NK_1_R-related fragment by an amide bond in the **D** region (**4.5**–**4.7**). A rather infrequent idea to develop ionic pairs (**4.8**–**4.10**) was pursued, too. In these, the fentanyl-related carboxylates were paired with aminium derivative (**4.11**, Scheme 4) related to **4.1**. 

The carboxylates (**4.2**–**4.4**) were synthesized (Scheme 4) by acylating **1.8** with appropriate cyclic anhydrides. The desired covalent hybrids (amides **4.5**–**4.7**) were then obtained by coupling the amine **4.11** following a typical peptide chemistry approach (carboxyl activation by a carbodiimide or a phosphonium salt). An interesting alternative based on succinisoimidium perchlorates chemistry was developed, too. In this route, the acids (**4.2**–**4.4**) were treated with acetic anhydride and perchloric acid to give isoimidium perchlorates (**4.12**). These were then reacted with a hydrochloride aminium **4.11** to yield the desired hybrids. Finally, the ionic pairs (**4.8**–**4.10**) were obtained by simple mixing the potassium salts of **4.2**–**4.4** and the hydrochloride aminium **4.11**. 

The obtained covalent hybrids turned out to have moderate MOR affinity (Table 3), with K_i_’s being 400 nM in the case of an ether derivative (**4.7**) or slightly greater than 100 nM in the cases of **4.5** and **4.6.** On the other hand, the ionic compounds exhibited MOR K_i_’s greater than 1 μM, what suggests that an acidic moiety in the region **D** of fentanyl is highly unfavourable to MOR binding. As to the NK_1_R binding, both the covalent and the ionic compounds had low nanomolar affinity, with K_i_’s ranging 6.8–44 nM. The compound **4.6** had the lowest K_i_ values in binding to both receptors.

Consistently with the affinity data, the hybrids had weak or very weak agonistic (and no antagonistic) activity at opioid receptors in isolated tissues. In the NK_1_R functional assays, they were found to antagonise the effects of SP, with **4.6** being the most efficient in this. As this analogue exhibited some moderate MOR affinity and agonism too, the authors concluded that **4.6** could serve as a lead compound. They pointed that elongation and other variations in the connecting spacer (e.g., insertion of a peptide fragment) will be a direction for further work.

## 5. Fentanyl-Based MTAs Targeting MOR and D_2_-like Dopamine Receptors

Other non-opioid GPCRs which are of interest as potential co-targets for multifunctional analgesics are D_2_-like dopamine receptors (D_2_-likeRs). This subfamily includes D_2_, D_3_ and D_4_ dopamine receptors. These proteins and MOR exhibit co-distribution in several parts of the brain [67]. There is in vitro evidence that suggests the existence of D_2_R-MOR and D_4_R-MOR heterodimers [68]. Moreover, there are reports on the cross-regulation of opioid and dopaminergic system, in particular in reward processes [69,70,71,72,73,74]. 

In the light of these facts, simultaneous targeting of MOR and D_2_-likeRs (as separate receptors or as heterodimers) may be a basis for innovative, nonaddictive analgesics. Qian et al. demonstrated feasibility of targeting MOR/D_2_-likeRs heterodimers by long molecules containing alkaloid MOR-related fragments (naltrexone, hydromorphone) [68]. Bonifazi et al. synthesized MOR-D_3_R bitopic/bivalent compounds in which opioid fragment was based on acyclic opioids [75]. 

**Figure 6:**
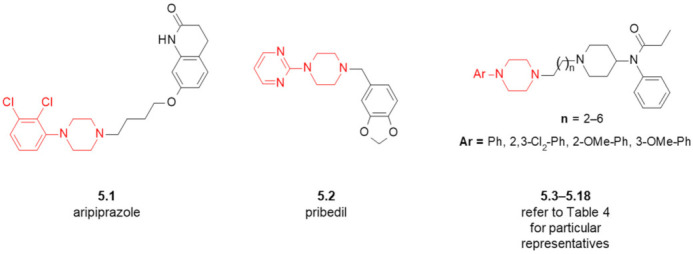
Structures of dopamine receptor ligands and of the designed fentanyl-based MOR/D_2_R MTAs. In red marked are fragments characteristic for D_2_R-ligands.

The possibility to employ a substructure of fentanyl (4-anilidopiperidine) in MOR/D_2_R multitarget ligands was investigated by Jevtić et al. [76,77]. The D_2_R pharmacophoric element to be incorporated was *N*-arylpiperazine which is present in D_2_R ligands such as aripiprazole (**5.1**, Figure 6) or pribedil (**5.2**). This element was installed (**5.3**–**5.18**) in region **B** of fentanyl structure by alkyl chains of variable length (2 up to 6 methylene units).

**Scheme 5:**
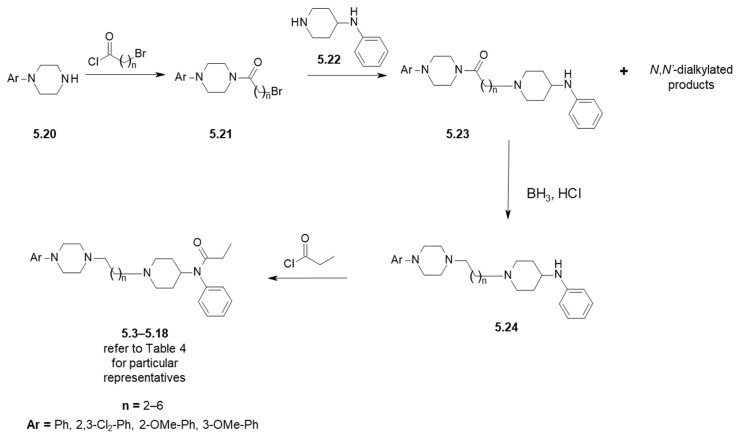
Preparation of fentanyl-based MOR/D_2_R ligands. See Table 4 for particular representatives.

The synthetic approach devised at first was intended to consist of two alkylations of secondary amines in piperazine and piperidine derivatives. In this approach, norfentanyl (**5.19**, Scheme S2) reacted with α,ω-bromochloroalkanes, but instead of desired linear products it was spiro-bicyclic quaternary ammonium salts that were formed (Scheme S2). In the alternative approach (Scheme 5), *N*-arylpiperazines (**5.20**) were subject to acylations with ω-bromoacyl chlorides, and the resulting bromides (**5.21**) served for *N*-alkylation of 4-anilinopiperidine (**5.22**). In the latter step, quite large amounts of *N*,*N*′-dialkylated products were also observed with the second alkylation taking place at the anilino nitrogen. After removing these impurities, borane reduction of the tertiary carboxamido group in **5.23** gave compounds **5.24** that were acylated with propionyl chloride to yield the designed compounds (**5.3**–**5.18**). 

The prepared analogues were tested in vitro for binding to dopamine receptors and in vivo for their antinociceptive activity (in rats, using tail-immersion test after ip injection). In the latter of the performed test, antinociceptive activity in doses up to 2 mg/kg was absent. Not necessarily does this exclude MOR affinity of the studied compounds, since as the authors noted themselves, physicochemical properties of the compounds or their metabolism could impair distribution into CNS. Hence, further research programmes based on these analogues require that MOR affinity is measured.

With regard to dopamine receptor binding, the studied analogues showed rather moderate affinity with K_i_ ranging from 594 nM to 8105 nM (Table 4). The best (submicromolar) affinities were observed for the shortest compounds (with three methylene units as a linker, n = 2, **5.3**–**5.5**). Elongation of the linker resulted in deterioration of binding strength (Figure S3A) so that none of the compounds with n > 2 exhibited submicromolar K_i_. The effect of substituents on the *N*-aryl ring seems non-additive to the effect of chain elongation (see Figure S3B–D and below for our QSAR analysis). When n = 2, 4 or 5, the following binding preference is found 2,3-Cl_2_-Ph > 2-OMe-Ph > Ph. On the other hand, for n = 3 or 6, analogues with unsubstituted phenyl have much better affinity than those with the substitutions present, and so the preference is Ph >> 2,3-Cl_2_-Ph ~ 2-OMe-Ph. This could suggest a binding mode switch with the length of the linker. Jevtić conducted preliminary docking analysis of a few analogues with the intent of explaining the observed D_2_R affinities [78]. An important observation is that while the arylpiperazine moiety resides deep in the orthosteric pocket, while the anilidopiperidine moiety is located in the extended binding pocket. The key polar interaction with Asp114 (expected for high affinity at D_2_R) is formed, but it may be of suboptimal geometry and for this reason, the D2R affinity is rather moderate. The obtained binding models might serve for further optimization of the affinities. 

As a side note, let us mention that an avenue that might deserve exploration is using fentanyl scaffold for designing compact (‘merged’) MOR/D_4_R multifunctional drugs. Fentanyl (**1.1**) has been recently shown to have almost no D_2_R (K_i_ = 21,000 nM) and no D_3_R binding (K_i_ = 26,200 nM), but some moderate, submicromolar affinity for D_4_R (K_i_ = 554 nM) [75]. 

## 6. Fentanyl-Based MTAs Targeting MOR and COX

Not only receptors but also enzymes are considered as targets for the MTAs, however these attempts (at least in combination with opioid receptors as co-targets) seem less frequent. There is a single report by Vardanyan et al. [23] on ligands designed to be MOR agonists and inhibitors of cyclooxygenases (COXs). COXs are enzymes involved in the production prostaglandins from arachidonic acid, and in this way, they participate in the inflammatory and pain reactions. Inhibition of COXs is the main mechanism of action for the non-steroidal anti-inflammatory drugs (NSAIDs) which are popular analgesic compounds with anti-inflammatory and antipyretic action. NSAIDs and opioids are sometimes used together in multimodal management of pain because of the purported synergistic effect [79], and in some markets available are fixed-dose opioid/NSAIDs combinations. Multitarget opioid receptors/COX-targeting analgesics could be in principle superior to these for the reasons of dosing convenience and pharmacokinetics.

**Figure 7:**
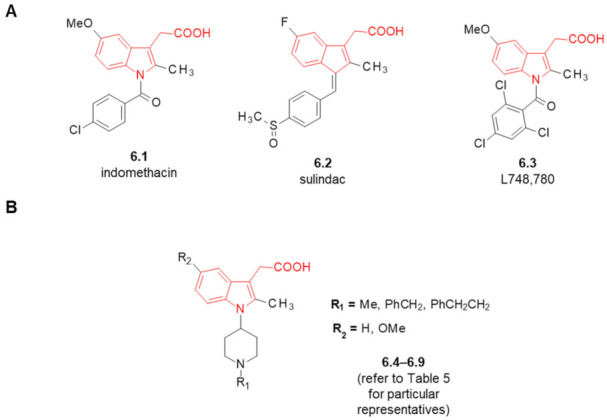
(**A**) Structures of COX inhibitors. (**B**) Structures of the designed fentanyl-based MOR/COX MTAs. In red marked is indolyl/indene acetic acid motif.

Vardanyan et al. [23] attempted creating such hybrids by combining fragments of fentanyl with the indolyl/indene acetic acid motif present in some NSAIDs, such as indomethacin (**6.1**, Figure 7A) sulindac (**6.2**) or L748,780 (**6.3**). The motif was to be melted into **C** and **D** region of fentanyl structure to give compounds (**6.4**–**6.9**).

**Scheme 6:**
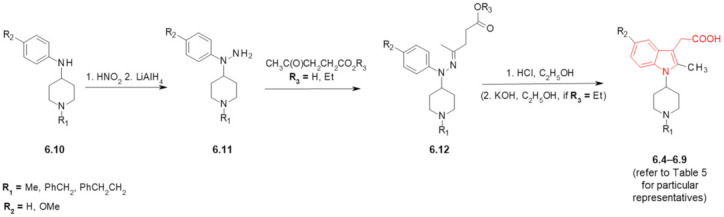
Preparation of fentanyl-based MOR/COX ligands. See Table 5 for particular representatives. In red marked is indolyl motif characteristic for COX inhibitors.

On the route to these analogues (Scheme 6), appropriate *N*-substituted 4-anilinopiperidines (**6.10**) were subject to nitrosylation with HNO_2_ and the resulting *N*′-nitroso derivatives were hydrogenated to obtain hydrazines (**6.11**). By condensation of these with levulinic acid or its esters, hydrazones (**6.12**) were formed which in the presence of HCl in ethanol converted to indole derivatives with the desired substitution pattern (**6.4**–**6.9**). 

Unfortunately, the expected dual activity was not confirmed in the biological assays (Table 5). The analogues exhibited very low opioid activity, as measured by assays in tissue preparations (GPI/LM/MP and MVD). For only one of them (**6.5**, R_1_ = PhCH_2_, R_2_ = H) micromolar IC_50_s were established (GPI/LM/MP ~ 5 µM, MVD ~ 1 µM). None of the compounds had antagonistic activity at MOR and DOR at 1 µM. 

Regarding the COX inhibition, the compounds tested at a concentration of 50 nM did not inhibit production of prostaglandin by COX-1 or COX-2. In line with the receptor/enzyme data, the compounds showed no in vivo antinociceptive activity in rat models of acute and chronic pain (10 µg, intrathecal).

The authors related the lack of opioid activity to conformational differences in fentanyl and the indole-incorporating derivatives (on comparing the crystal structures of fentanyl **1.1** and of an ester derivative of **6.6**). 

## 7. Fentanyl-Based MTAs Targeting MOR and FAAH/MAGL Hydrolases

Other enzymes that are relevant to the subject of this review are fatty acid amide hydrolase (FAAH) and monoacylglycerol lipase (MAGL). FAAH and MAGL are hydrolases that participate in the catabolism of endocannabinoids. Their inhibition increases levels of endogenous cannabinoids and in this way it may bring antinociception [80]. Indeed, blocking of FAAH or MAGL was demonstrated to result in analgesic activity in different pain models [81,82]. Several FAAH and MAGL inhibitors were advanced to clinical trials (in indications related to pain, but not only thereto), but as of today it did not result in approved drugs [83]. Both enzymes attract attention in the multitarget approach, too. 

**Figure 8:**
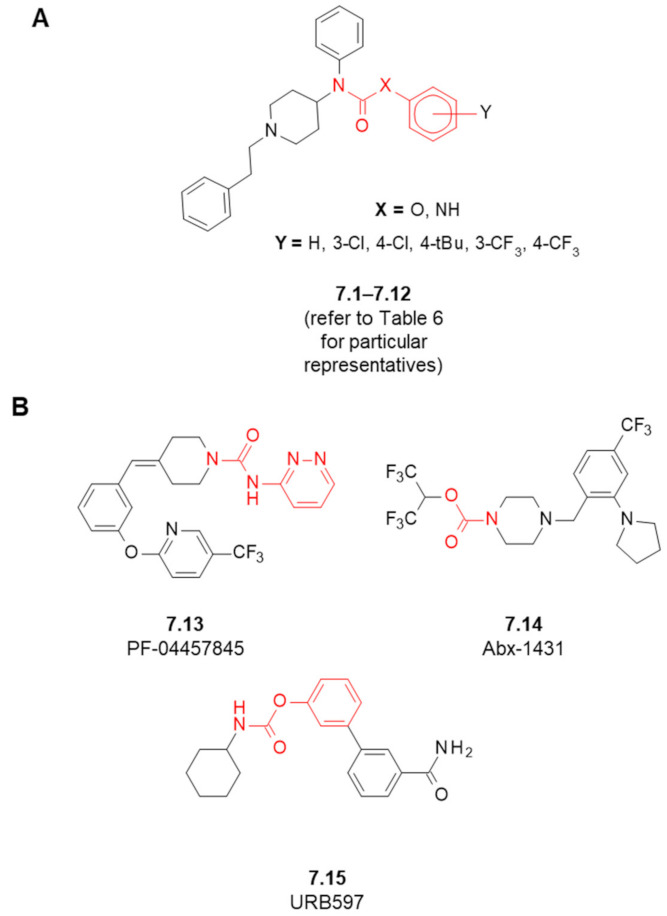
(**A**) Structures of the designed fentanyl-based MOR/FAAH/MAGL MTAs. (**B**) Structures of selected FAAH/MAGL inhibitors. In red marked is carbamate/urea substructures.

**Scheme 7:**
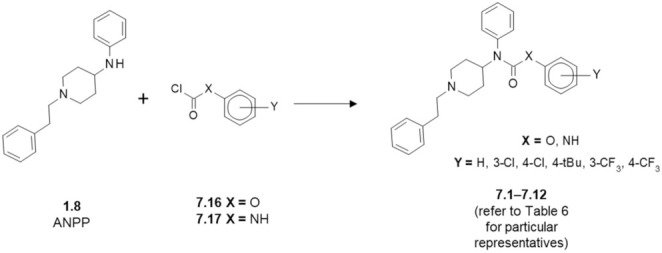
Synthesis of fentanyl-based MOR/FAAH/MAGL ligands.

Monti et al. proposed two series of fentanyl-related analogues **7.1**–**7.12** in which *N*-arylurea or *O*-arylcarbamate substructures were melted in region **D** of the fentanyl structure (Figure 8A) [24]. Both these motifs are present in either FAAH or MAGL inhibitors [84] (e.g., **7.13**–**7.15**, Figure 8B). The synthesis of fentanyl-derivatives **7.1**–**7.12** (Scheme 7) was accomplished by reacting 4-anilino-*N*-phenethylpiperidine (**ANPP**, **1.8**) with appropriate chloroformates (**7.16**) or *N*-arylcarbamoyl chlorides (**7.17**). 

The MOR affinities of the synthesized compounds were at best moderate (Table 6). In no case was IC_50_ better than 500 nM. The best binding derivative, undecorated urea **7.7**, had IC_50_ = 516 nM. Slightly worse values were found for **7.3**, (X = O, Y = 3-Cl) **7.8**, **7.9** (X = NH, Y = 4-Cl or 3-Cl). A dramatic deterioration in MOR affinity was found upon introduction of 4-tBu substituent in the urea series (**7.10**) leading to IC_50_ > 20,000 nM. No general SAR trend regarding MOR affinity can be found in this series, except perhaps for stating that the effect of the substituent is not additive to the effect of urea/carbamate linker (Figure S4). According to the modelling performed by Monti et al. [24], the analogues **7.1**–**7.12** bind to MOR in a manner only partially matching the binding mode of fentanyl which could explain moderate affinity and different functional properties.

Regarding the enzymatic activity (Table 6), the analogues did not affect the activity of either FAAH or MAGL. Only trace signs of inhibition were found at concentration as high as 10 μM. Moreover, the authors examined if compounds **7.2** and **7.3** could bind to DOR, KOR and CB_1_R, in all cases finding IC_50_ values in the micromolar ranges. 

In the functional assay ([^35^S]GTPγS binding), all the novel analogues turned out to be inverse agonists properties, reducing G-protein basal activity (efficacy in the range 80–100%, potency in the range 3–5 μM). The effect was not reversed by the opioid antagonist naloxone. All in all, this suggests that the studied derivatives are active against some other, non-opioid molecular target of the GPCR family. Interestingly, two compounds (**7.2** and **7.3**) were found to have some antinociceptive activity in vivo in hot plate test in mice, but only at high doses, and with no apparent relationship to opioid receptor affinity or to enzymatic inhibition.

## 8. Fentanyl-Related MTAs Targeting MOR and σ_1_R

As the last of the ‘second’ targets for fentanyl-based MTAs, we shall discuss the σ_1_ receptor (σ_1_R). Despite its name, the σ_1_ receptor is not a ‘typical’ receptor, but it is thought to be rather a ‘ligand-operated’ chaperone [85]. σ_1_R stabilizes proteins of endoplasmic reticulum but also regulates (directly or indirectly) ion channels [86], kinases and receptors, including some GPCRs such as the dopamine receptors [87] or μ-opioid receptor [88]. For being involved in many physiological and pathological processes [89,90], σ_1_R was proposed as a therapeutic target for the treatment of inter alia schizophrenia, depression, drug addiction, neurodegenerative diseases or neuropathic pain [91]. 

As to the latter, it was shown that σ_1_R antagonists do not have antinociceptive action in classical models of acute nociception [92,93,94], however they inhibit pain in sensitizing pain models [95,96,97,98]. Most importantly, σ_1_R antagonists were found to enhance antinociceptive action of classical opioids [99] but without exacerbating their side-effects (tolerance, dependence, constipation) [100]. For these reasons, σ_1_R antagonists were proposed not only as a stand-alone treatment against neuropathic pain but also as adjuvants for opioid therapy [87,100,101]. 

A closely related idea to combine MOR agonist and σ_1_R antagonist activities in one molecule was probably expressed for the first time in the early 2010s in a few patent applications by ESTEVE Laboratories [102,103,104]. In the scientific literature, per our knowledge, a first suggestion along these lines was made by Prezzavento et al. [105]. These authors showed that phenazocine enantiomers bind to both MOR and σ_1_R with high affinity and that their antinociceptive action is associated with both these receptors. Hence, they suggested that phenazocine structure might be a scaffold for developing dual MOR agonist/σ_1_R antagonist ligands. In the past very few years (from 2019 onwards) there appeared several papers describing efforts based on the concept of dual MOR/σ_1_R ligands, nicely summarized in a recent review [22].

### 8.1. Affinity of Fentanyl Analogues for σ_1_R

**Figure 9:**
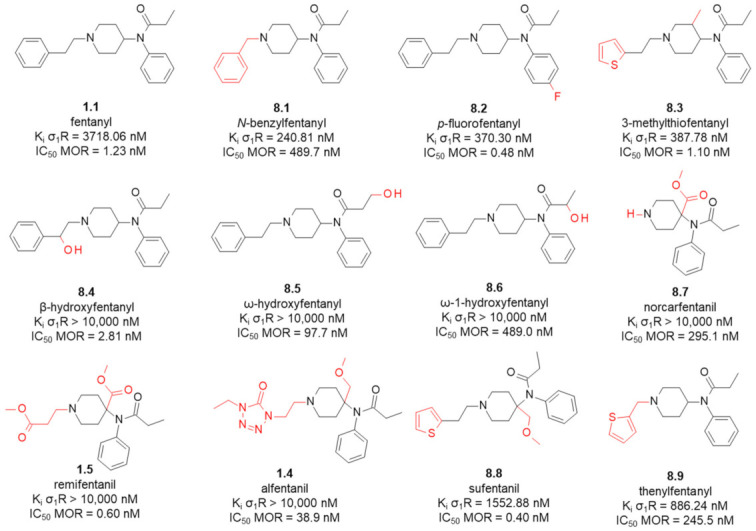
MOR and σ_1_R affinities of fentanyl and analogues. In red marked are structural differences compared to the parent compound. Data taken from [106] (σ_1_R, competitive assays done in guinea pig brain membrane homogenates, [^3^H]-(+)-pentazocine as radioligand) and from [109] (MOR, competitive assays done in rat brain membrane homogenates, 0.5 nM [^3^H]DAMGO as radioligand).

That fentanyl might be a basis for such dual ligands was one of the conclusions in a 2019 paper of ours [106]. In that study we assayed fentanyl and its 11 commercially available analogues for σ_1_R affinity (Figure 9). Our initial interests in the fentanyls’ affinities for σ_1_R were rather remote from typical medicinal chemistry, but instead we wanted to see if σ_1_R affinity could be an important ingredient of fentanyls’ secondary pharmacology.

In agreement with the previous reports [107,108], fentanyl (**1.1**) showed a rather low σ_1_R affinity with K_i_ = 3718 nM. Interestingly however, minor structural modifications to the parent structure result in submicromolar affinities. For example, *N*-benzylfentanyl (**8.1**, Figure 9) which is different from the parent by having one methylene unit -CH_2_- less in the **B**-region (*N*-chain), has K_i_ = 240 nM. *p*-fluorofentanyl (**8.2**) which differs just by having a fluorine atom instead of a hydrogen in the region **C**, exhibits binding with K_i_ = 370 nM. Similar affinity is found for 3-methylthiofentanyl (**8.3**. K_i_ = 387.78 nM) that has a methyl group in the piperidine position C3 and a 2-thienyl ring (instead of the phenyl ring) in the *N*-chain (region **B**). The analogues with the 4-axial substitution in the piperidine ring exhibit none or low affinity for σ_1_R (**1.4**, **1.5**, **8.7**, **8.8**). Similarly, introduction of a hydroxyl group into the regions **B** or **D** gives compounds without σ_1_R affinity (**8.4**, **8.5**, **8.6**).

According to modelling of the interactions between σ_1_R and selected fentanyls (docking and molecular dynamics), both *N*-benzylfentanyl (**8.1**) and *p*-fluorofentanyl (**8.2**) adopt a binding mode that shares the main features with the binding mode of a high-affinity ligand 4-IBP as found in the 5HK2 crystal structure [110], these being:

- ionic interaction of protonated piperidine’s nitrogen with Glu172,
- direction of the anilide’s ring towards α4 and α5 helices of the receptor,
- positioning of the *N*-substituent towards the bottom of β-barrel (close to Asp126).

Apart from the above mentioned, single ionic interaction, the remaining ligand-receptor contacts seen in the simulations were of apolar character. Based on these computations, we were able to provide reasonable explanations for the rest of the observed affinities. 

Comparing the σ_1_R binding data to MOR affinities (Figure 9), it is seen that while most of the studied analogues turned out to be MOR-selective, *N*-benzylfentanyl (**8.1**) may be considered a balanced binder to both receptors (or with some minor preference for σ_1_R). As such, this very analogue could be a starting point in the search for mixed MOR/σ_1_R ligands. 

### 8.2. 1-Oxa-4,9-diazaspiro[5.5]undecane Derivatives

**Figure 10:**
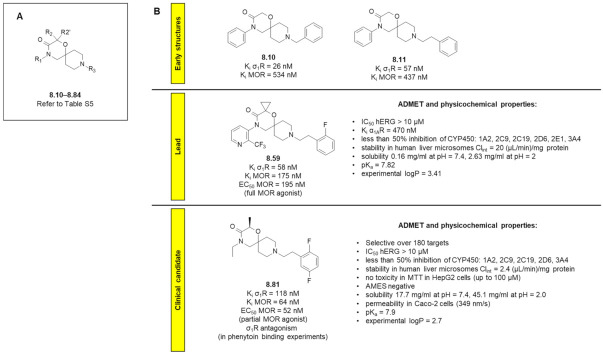
(**A**) General structure of compounds **8.10**–**8.84**, (**B**) Structures of key compounds in the programme with some of their pharmacological properties.

A very successful med-chem programme focused on dual μOR agonists/σ_1_R antagonists was recently described by García et al. (of ESTEVE Pharmaceuticals SA) [111,112]. In their contributions the team disclosed 1-oxa-4,9-diazaspiro[5.5]undecane (Figure 10) derivatives with the desired dual activity. Although the discovery of these compounds was not directly inspired by fentanyl’s structure, some not so remote a similarity between fentanyl (or *N*-benzylfentanyl) and the early structures in this programme (e.g., **8.10** and **8.11**) is obvious to chemist’s eyes (Figure 10B). The initial compounds were designed as a result of merging 3D-pharmacophore models of both receptors (based on morphine for μOR and based on a model by Laggner et al. [113] as well as on the in-house SAR data [114] in the case of σ_1_R).

Almost 80 analogues (**8.10**–**8.84**, see Table S5 for structures and affinities) were reported to have been obtained and tested [111,112], out of which about a half exhibited K_i_ < 100 nM for MOR and about 50 of them showed K_i_ < 100 nM for σ_1_R. Many examples had single-digit nanomolar K_i_’s either for one of the receptors or for both. The issues that were mainly fought with during the SAR exploration was the propensity of these structures to interact with hERG channels and α_1A_-adrenergic receptors (α_1A_R; associated with cardiac toxicity). Increasing polarity (decreasing lipophilicity) of the structures was rather unsuccessful in coping with these liabilities, and additionally it gave potency losses at both targets of interest. R_2_ substitution with small alkyl groups was very favourable to the binding at primary targets. The preferred stereochemistry at this position (mainly due to MOR affinity) was *R* (compare for example **8.13** vs. **8.14**, **8.67** vs. **8.68**, **8.81** vs. **8.84)** For the R_3_ group, it was established that an arylethyl substituent is required for dual affinity. The later compounds drove structurally away off resembling fentanyl (**8.60**–**8.84**) [112]. The key step in SAR campaign was elimination of the aryl ring in the 4-position, since in this way it was possible to get rid of α_1A_R activity.

Important to note, as the lead compound (**8.59**) [112], the authors chose not the best opioid binder among **8.10**–**8.59**, but a compound with balanced affinities for the desired targets (MOR K_i_ = 175 nM, σ_1_R K_i_ = 58 nM). This was justified by the expectation that the best benefit-to-risk ratio would be obtained upon combining σ_1_R antagonism with weak/partial MOR agonist: “*efficacy would result from σ_1_R antagonism-mediated maximization of modest opioid effect whereas side effects would rely on such nonpotentiated baseline opioid component*” [112].

The lead **8.59** was a full MOR agonist (in vitro), showed moderate α_1A_R binding (K_i_ = 470 nM), no hERG inhibition and selectivity in the selectivity panel. It did also have favourable physicochemical properties and good ADMET data (Figure 10B). In vivo, the compound was shown to be active in acute pain model (mouse paw pressure pain test, ip administration) with ED_50_ = 15 mg/kg, albeit the compound was less potent than the reference oxycodone (per os, po). On the other hand, the authors showed that **8.59** produced less inhibition of the intestinal transit in mice than the reference oxycodone (at equianalgesic doses: 20 mg/kg ip **8.59** vs. 10 mg/kg po oxycodone). Upon intraplantar administration (ipl; 25 μg), **8.59** showed local analgesic effect (paw pressure test in mice) which was abolished by a σ_1_R agonist PRE-084, that showing a hint in favour of the double mechanism of action.

The lead optimization work eventually resulted in EST73502 (**8.81**, Figure 10B) [112]. This compound turned out to have good and balanced on-target affinities (σ_1_R K_i_ = 118 nM; MOR K_i_ = 64 nM) and to be very selective (against 180 molecular targets; hERG inhibition > 10 μM; low CYP involvement). It did also show favourable physicochemical and ADMET properties (Figure 10B). EST73502 was effective in vivo in the acute pain model (paw pressure test in mice) after oral administration, showing a dose-dependent analgesic effect (64% of MPE) with ED_50_ = 14 mg/kg. The contribution of σ_1_R to the analgesic activity of EST73502 was confirmed by the observation that subcutaneous (sc) administration of PRE-084 (σ_1_R agonist) diminished the level of antinociceptive effect. The effect was fully abolished with sc administration of MOR antagonist naloxone.

EST73502 (**8.81**) was also tested in vivo in the chronic pain model (partial sciatic nerve ligation in mice) using the von Frey test. The compound (5 mg/kg, ip) was effective over 23 days of the experiment, at the level similar to that produced by oxycodone (1.25 mg/kg, ip). Importantly, contrary to oxycodone, EST73502 (**8.81**) did not produce opiate withdrawal signs (upon administration of naloxone). The dual analgesic showed also less inhibition of the intestinal transit in mice compared to oxycodone (at equianalgesic doses). With all these favourable characteristics, EST73502 (**8.81**) was nominated a clinical candidate. As of 2021, the Phase-I study was announced. Overall, this case demonstrates great potential of the MOR/σ_1_R dual ligands concept.

Of note, ESTEVE disclosed also a few sets of dual MOR/σ_1_R ligands based on spiroisoquinoline-1,4′-piperidine [115], spiroisoquinoline-4,4′-piperidine [116], amide [117] or piperidinylalkylamide [118] motifs. In some aspects of their structures, all these derivatives bear resemblance to the structure of fentanyl. In particular, many of piperidinylalkylamides in reference [118] are structurally related to *N*-benzylfentanyl (**8.1**, Figure 9).

### 8.3. Amide Derivatives with Piperidine in Their Structures

**Figure 11:**
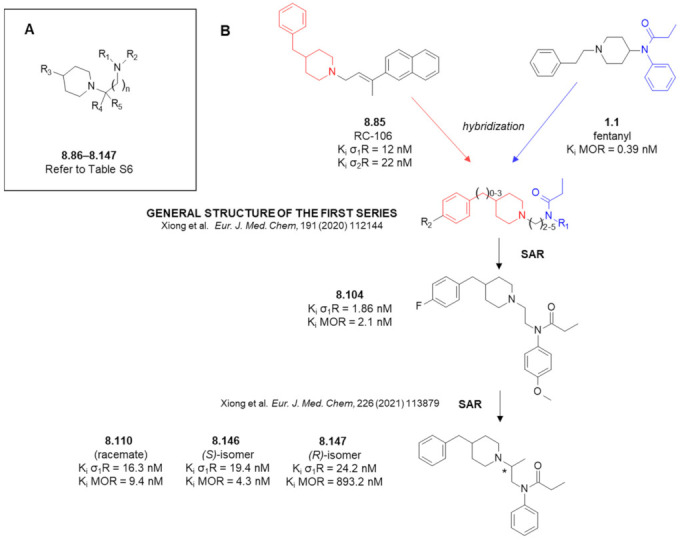
(**A**) General structure of compounds **8.86**–**8.147**. (**B**) Design principle and key compounds in the programme with some of their pharmacological properties. In red and blue marked are fragments subject to hybridization.

Xiong et al. successfully designed dual MOR/σ_1_R ligands by molecular hybridization. These authors combined (Figure 11) *N*-phenylpropionamide fragment (of fentanyl structure) with the 4-benzylpiperidine (of RC-106 **8.85**, a pan-sigma ligand) [119]. In the works of theirs [119,120], over 60 dual ligands (**8.86**–**8.147**, Figure 11 and Table S6) were reported to have been synthesized and tested (Table S6). Around a half of the published examples had K_i_ < 100 nM for either of the receptors of focus. For nine analogues, K_i_ values were below 30 nM for both receptors.

Of the key SAR findings, with respect to R_1_ and R_2_ groups (Figure 11), the *N*-phenylpropanamide motif (optimally with *para*-fluoro or *para*-methoxy substitution at the ring) is most advantageous for the target affinities. The optimal spacing between the piperidine ring and the amide was afforded by ethylene linker. As to the R_3_ group, the best affinities were found for benzyl or substituted benzyl moieties.

Of the first series of analogues [119], compounds **8.104** (Figure 11B) and **8.108** (Table S6) were advanced to more detailed in vitro and in vivo testing. These compounds were found to be selective against a set of a few receptors (σ_2_R, 5-HT_1A_, 5-HT_2A_, histamine H_3_ receptor, cannabinoid CB_1_ and CB_2_ receptors; less than 50% binding at 1 μM). Their median lethal doses (**8.104**, LD_50_ = 396.7 mg/kg; **8.108**, LD_50_ = 415.8 mg/kg; sc injection) were higher than that of a reference σ_1_R antagonist (compound S1RA, LD_50_ = 357.4 mg/kg, sc). In vivo, in the formalin test in mice, both **8.104** and **8.108** were found to exhibit antiallodynic activity (sc pretreatment with 50 mg/kg before formalin injection; half-maximal effective dose, ED50 for compound **8.104** in Phase II was 15.1 mg/kg). Additionally, this analogue was examined in the chronic constriction injury (CCI) model of neuropathic pain (in rats; von Frey test, day 15 after surgery). Here, 25 mg/kg (sc) of compound **8.104** was found equianalgesic to 50 mg/kg (sc) of S1RA. ED_50_ of compound **8.104** was 44.14 mg/kg.

As a result of SAR work around compound **8.104** [120], racemic compounds **8.110** (Figure 11B) and **8.131** (Table S6) were identified as very promising. Enantiomers of **8.110** were synthesized and assayed (**8.146** and **8.147**, Figure 11B). Interestingly, there was a notable stereoselectivity in case of MOR affinity in favour of *S*-configuration (**8.146**), but σ_1_R affinities were at a similar level for both isomers. The MOR-agonist/σ_1_R antagonist profile of compounds **8.110**, **8.131** and **8.146** was confirmed in functional testing. The compounds were also found selective (less than 50% binding at 10 μM to KOR, DOR, σ_2_R, 5-HT_1A_R, 5-HT_2A_R, H_3_R, serotonin transporter and noradrenaline transporter) and safe (acute toxicity after sc injection; the best LD_50_ = 271.6 mg/kg found for **8.146**).

The compound **8.146** was also found to be an effective analgesic in mice in the acetic acid-induced writhing test (ED_50_ = 0.47 mg/kg) and in the formalin test (acute and chronic pain; ED_50_ = 0.32 mg/kg). In the latter test, 1 mg/kg (sc) of **8.146** was similarly effective in Phase I to the maximally effective dose of 0.05 mg/kg fentanyl (sc). In the hot-plate test, 3 mg/kg **8.146** (sc) was equianalgesic to fentanyl 0.1 mg/kg (sc) while ED_50_ was found to be 1.0 mg/kg. In the Von Frey test (neuropathic pain CCI-model) 0.3 mg/kg was equianalgesic to 0.05 mg/kg of fentanyl and its ED_50_ = 0.48 mg/kg.

Compound **8.146** was also investigated as to the typical opioid-side effects and compared to fentanyl (sc injections at equianalgesic doses of 1 mg/kg and 0.05 mg/kg, for **8.146** and fentanyl, respectively). It was found that **8.146** did not produce conditioned place preference, did not depress the respiratory rate (whole body plethysmography), did not induce physical dependence (naloxone-induced withdrawal) and did not have any significant effect on exploratory locomotor activity. On the contrary, fentanyl showed all these undesired characteristics despite a much lower dose. Additionally, favourable pharmacokinetic properties of Compound **8.146** were found after 1 mg/kg sc administration (t_1/2_ = 1.71 h, T_max_ = 0.25h, C_max_ = 214 ng/mL). Given all these favourable properties, the compound **8.146** was described as a potential candidate drug for treating neuropathic pain, with further studies of this compound heralded.

### 8.4. Remark on σ_2_R

Let us add here that a direction worth examination is if fentanyl-based compounds could bind another σ-binding site, the σ_2_ receptor (σ_2_R, transmembrane protein 9, TMEM97 [121]). It seems probable that they do, since many σ_1_R ligands have appreciable affinity for σ_2_R, too. There is also a prediction from a QSAR model based a large dataset of σ_2_R ligands that fentanyl would bind σ_2_R with K_i_ in middle nanomolar range [122]. If confirmed, this could hypothetically open way to mixed opioid/σ_2_R ligands utilizing the fentanyl scaffold. Application of σ_2_R ligands in the therapy of cancers and neurological, inflammatory and autoimmune disease has been proposed [123].

## 9. Outlook

Opioid/non-opioid multitarget analgesics are a promising approach towards obtaining more effective and safer drugs against pain, including chronic pain with neuropathic components. A key consideration in search for MTAs is the choice of pharmacophores to be used and of the manner in which they are brought together in one molecule. In this review we discussed the attempts to create opioid/non-opioid MTAs that utilized fentanyl structural elements as opioid pharmacophores.

In most of the considered cases, the auxiliary pharmacophores were introduced by fusing both parts ‘side-to-side’ or by separating them by the means of a linker. While conceptually simple, such approaches are rarely successful with just one ‘shot’. The structure of fentanyl is rather compact and non-redundant and any replacements or deletions may lead to deterioration or ablation of opioid activity. At the same time, it is not easy to introduce the second type activity into the structure. Moreover, structural optimization is difficult due to the fact that MOR affinity trends can be antiparallel to those of the auxiliary target. Intriguingly, even if opioid affinity is preserved (at least to some extent), not necessarily follows the functional activity and even inversion of function (agonist into antagonist) can occur.

Which further directions are worth exploration? In none of the ‘fusing’ or ‘linking’ attempts were employed the structural modifications known to improve MOR affinity of fentanyls, e.g., α-methyl, β-hydroxyl, 3-methyl, 4-methoxymethyl, 4-carboxymethyl etc. In particular, the two latter substitutions deserve examination in MTAs, since they are known to produce very potent MOR ligands. On the other hand, it cannot be guaranteed that these substitutions would be compatible with the requirements of the auxiliary molecular target For example, it seems that 4-axial substitution at the piperidine ring negatively affects σ_1_R binding [106]. A certain problem with 4-axial substituted fentanyl analogues is that they require multi-step and rather low-yielding syntheses. Notably however, some progress in their syntheses have been reported in several past years [124,125,126,127]. Moreover, in the field of mixed opioid ligands, there is an interesting recent example, in which carfentanil (**1.2**) fragments were hybridized with peptide dermorphine analogues to yield potent analgesics with improved properties [128].

Yet other direction that have not been tried so far is attaching the linker directly to the piperidine ring (e.g., in positions 3 or axial 4 in region **A**) without removal or modification of the remaining fentanyl elements. This is likely to be synthetically demanding but several valuable strategies that might enable it have been described [129,130,131].

Future attempts should also benefit from applying molecular modelling and structure-based approaches. Recent years have witnessed major progress in GPCR structural biology. This has enabled wider application of structure-based approaches in GPCR ligand discovery [132,133]. For the design of MTAs, it is vital that four MOR structures (Table S7) are now available in the PDB database [134,135,136]. Of particular importance, interactions of fentanyl with MOR were subject of several recent studies that used docking, molecular dynamics and other modelling techniques [109,137,138,139,140,141,142,143]. A useful tool for interpretation of MOR ligands’ SAR, based on template alignment, modelling have been devised, too [144]. It is also for many non-opioid GPCRs related to pain (including those discussed in this paper) that the structures have been recently solved (Table S8, refer to the GPCRdb service for an up-to-date and comprehensive list [145]). Of late, structural insights have become available also for binding of ligands to the σ-receptors [110,146,147] (Table S9) All these, along with extensive SAR data gathered over the years, might be expected to expedite opioid/non-opioid MTAs’ design.

## Figures and Tables

**Table 2:** Affinities and antagonistic properties of fentanyl-based MOR/CB_1_R ligands and of reference compounds for the intended molecular targets. Data are from [50] unless specified otherwise.

	Structure (Refer to Figure 4)	Affinity [K_i_ (μM)] ^1^		Antagonistic Properties (an Agonist’s EC_50_ in μM, [^35^S]GTPγS Binding Assay ^2^, in Presence of 10 μM of a Tested Compound)	
Compound	X	MOR ^3^	CB_1_R ^4^	Fentanyl (Alone) EC_50_ = 0.28 ± 0.04 μM	WIN55,212-2 (Alone) EC_50_ = 1.1 ± 0.22 μM
**Fentanyl (1.1)**	-	0.003 ± 0.001	-	-	-
**Rimonabant (3.1)**	-	0.20 ± 0.12 ^5^	0.004 ± 0.002	-	21 ± 3
**Naloxone**	-	-	-	456 ± 60	1.3 ± 0.17
**3.2**	-(CH_2_)_3_-	3.81 ± 0.39	0.19 ± 0.07	-	-
**3.3**	-(CH_2_)_4_-	1.23 ± 0.43	0.57 ± 0.20	24 ± 5	33 ± 8
**3.4**	-(CH_2_)_5_-	0.17 ± 0.10	>10	-	-
**3.5**	-(CH_2_)_6_-	0.30 ± 0.06	2.29 ± 1.86	33 ± 2	21 ± 2
**3.6**	-(CH_2_)_7_-	6.54 ± 0.95	0.70 ± 0.57	3 ± 1	16 ± 2
**3.7**	-(CH_2_)_8_-	1.24 ± 0.79	3.99 ± 1.37	-	-
**3.8**	-(CH_2_)_9_-	0.11 ± 0.06	>10	-	-
**3.9**	-(CH_2_)_12_-	6.90 ± 1.58	>10	-	-
**3.10**	mPh	1.25 ± 0.67	>10	-	-
**3.11**	mXyl	1.02 ± 0.25	>10	-	-
**3.12**	bcHex	0.66 ± 0.37	2.06 ± 0.60	-	-

^1^ K_i_, inhibition constant (μM) with standard error of the mean, ^2^ [^35^S]GTPγS functional assays on cortical membranes of post mortem human brain, ^3^ competitive assays done in membrane preparations of post-mortem human prefrontal cortex, 2 nM [^3^H]DAMGO as radioligand, ^4^ competitive assays done in membrane preparations of post-mortem human prefrontal cortex, 1 nM [^3^H]CP55,940 as radioligand, ^5^ note also interesting contributions on the influence of rimonabant on opioid receptors in references: [54,55,56].

**Table 3:** Receptor affinities and functional data for fentanyl-based opioid/NK_1_R MTAs. Data from [65].

	Structure	Affinity [K_i_ (nM)] ^1^		Functional Tests (Inhibition of the Contractile Response Generated by Electrical Stimulation)		
	X (Refer to Figure 5)	MOR ^2^	NK_1_R ^3^	MOR (GPI/LM/MP ^4^)	DOR (MVD ^5^)	NK_1_R (GPI/LM/MP ^4^)
				Opioid Agonism, Inhibition of Contraction Height (% at 1 μM or IC_50_ in nM)		Antagonism of SP Action ^6^ [K_e_ ± SEM (nM)] (at 1 μM)
**Covalently linked compounds**						
**4.5**	-	130	13	410 ± 42 nM	14%	240 ± 39
**4.6**	-CH_2_-	120	6.8	55 ± 12 nM	13%	21 ± 4.3 (at 100 nM)
**4.7**	-O-	400	31	30%	30%	480 ± 12
**Ionic pairs**						
**4.8**	-	>10,000	21	1.8%	4.3%	210 ± 42
**4.9**	-CH_2_-	3900	44	19.5%	19.8%	500 ± 130
**4.10**	-O-	1300	23	11%	17.1%	490 ± 68

^1^ K_i_, inhibition constant, ^2^ competitive assays using [^3^H]DAMGO as radioligand, done in membrane preparations from cells expressing human MOR, ^3^ competitive assays using 0.5 nM [^3^H]-Substance P as radioligand, done in membrane preparations of CHO cells stably transfected with human NK_1_R, ^4^ longitudinal muscle/myenteric plexus (LM/MP) of the guinea pig ileum (GPI), ^5^ isolated mouse vas deferens (MVD), ^6^ longitudinal muscle/myenteric plexus assay in presence of Substance P.

**Table 4:** Affinity fentanyl-based MOR/D_2_R MTAs. Data taken from [77].

Compound	Ar (Refer to Figure 6)	n	D_2_R Receptor Binding, [K_i_ (nM)] ^1^
**5.3**	Ph	2	869
**5.4**	2-OMe-Ph	2	800
**5.5**	2,3-Cl_2_-Ph	2	594
**5.6**	Ph	3	1357
**5.7**	2-OMe-Ph	3	7992
**5.8**	3-OMe-Ph	3	n/d ^2^
**5.9**	2,3-Cl_2_-Ph	3	6956
**5.10**	Ph	4	7083
**5.11**	2-OMe-Ph	4	4436
**5.12**	2,3-Cl_2_-Ph	4	2376
**5.13**	Ph	5	8105
**5.14**	2-OMe-Ph	5	3778
**5.15**	2,3-Cl_2_-Ph	5	1500
**5.16**	Ph	6	1853
**5.17**	2-OMe-Ph	6	5454
**5.18**	2,3-Cl_2_-Ph	6	5326

^1^ K_i_, inhibition constant, competitive radioligand binding assay with 0.2 nM [^3^H]-spiperone as radioligand, in preparations of rat caudate nuclei synaptosomal membranes, ^2^ n/d—not determined.

**Table 5:** Opioid activities of fentanyl-based MOR/COX MTAs. Data from [23].

Compound	Structure (Refer to Figure 7)		Functional Tests (Inhibition of the Contractile Response Generated by Electrical Stimulation)	
			DOR (MVD ^1^)	MOR (GPI/LM/MP ^2^)
	R_1_	R_2_	Opioid Agonism, Inhibition of Contraction Height (% at 1 μM or IC_50_ in nM)	
**6.4**	Me	H	17.9%	0.7%
**6.5**	PhCH_2_	H	IC_50_ = 1266 ± 355 nM	IC_50_ = 5164 ± 2043 nM
**6.6**	PhCH_2_CH_2_	H	19.5%	3.1%
**6.7**	Me	OMe	2.8%	0%
**6.8**	PhCH_2_	OMe	8.3%	3%
**6.9**	PhCH_2_CH_2_	OMe	0%	6%

^1^ isolated mouse vas deferens (MVD), ^2^ longitudinal muscle/myenteric plexus (LM/MP) of the guinea pig ileum (GPI).

**Table 6:** Biological data for the designed fentanyl-based MOR/FAAH/MAGL ligands for the intended molecular targets. Data are from [24].

	Structure (Refer to Figure 8)			Maximal Inhibition at 10 μM [%]	
Compound	X	Y	MOR Affinity [IC_50_ (nM)] ^1^	FAAH ^2^	MAGL ^3^
**fentanyl (1.1)**	-	-	5.99	n/d ^4^	n/d
**7.1**	O	-	1442	0	0
**7.2**	O	4-Cl	2180	0	0
**7.3**	O	3-Cl	654.2	1.90	0
**7.4**	O	4-tBu	1830	10.2	0.74
**7.5**	O	4-CF_3_	2657	7.40	0
**7.6**	O	3-CF_3_	4093	7.40	0.41
**7.7**	NH	-	516.8	7.25	0
**7.8**	NH	4-Cl	665.1	8.26	3.50
**7.9**	NH	3-Cl	658.9	9.76	7.32
**7.10**	NH	4-tBu	23,050	5.82	1.28
**7.11**	NH	4-CF_3_	4031	7.49	0
**7.12**	NH	3-CF_3_	1204	9.16	2.97

^1^ competitive assays done in rat brain membrane homogenates, 1 nM [^3^H]DAMGO as radioligand, IC_50_, half-maximal inhibitory concentration, ^2^ inhibition of enzymatic hydrolysis of anandamide measured in rat brain membranes, ^3^ inhibition of enzymatic hydrolysis of arachidonoyl-glycerol measured in cytosolic fraction from COS-7 cells, ^4^ n/d—not determined.

## Supplementary Materials

Supplementary materials can be found at https://www.mdpi.com/article/10.3390/ijms23052766/s1. References [148,149,150,151,152,153,154,155,156,157,158,159,160,161,162,163,164,165,166,167,168] are cited in the supplementary materials.
