# Supplementary material for: Fentanyl Structure as a Scaffold for Opioid/Non-Opioid Multitarget Analgesics
Source: Int J Mol Sci. 2022 Mar 2;23(5):2766. doi: 10.3390/ijms23052766 (PMC8910985; doi:10.3390/ijms23052766)
Supplement: Supplementary file 1 [file ijms-23-02766-s001.zip › ijms-1614480-supplementary.pdf]

Supplementary Materials

to:

Fentanyl structure as a scaffold for opioid/non-opioid multitarget analgesics

by

Piotr F. J. Lipiński [1,*], Joanna Matalińska[1]

[1] Department of Neuropeptides, Mossakowski Medical Research Institute Polish Academy of Sciences, Pawińskiego 5, 02–106 Warsaw, Poland; plipinski@imdik.pan.pl, jmatalinska@imdik.pan.pl

* Correspondence: plipinski@imdik.pan.pl.

Table of contents:

Scheme S1. Initial synthetic steps towards compounds **2.6** and **2.7**. SM-2
Figure S1. Correlational analysis of affinity data regarding fentanyl-related MOR/I2-IBS ligands. SM-3
Figure S2. Correlational analysis of affinity data regarding fentanyl-related MOR/CB_1R ligands. SM-4
Scheme S2. Reaction of norfentanyl (**5.19**) with α,ω-bromochloroalkanes. SM-5
Figure S3. Correlational analysis of affinity data regarding fentanyl-related MOR/D_2-likeR ligands. SM-5
The QSAR Equations based on indicator variables for MOR/D_2R ligands. SM-6
Table S1. Indicator variables matrix for compounds **5.3** – **5.18**. SM-6
Table S2. Indicator variables and their coefficients (for Eq S1). SM-6
Table S3. Indicator variables and their coefficients (for Eq S2). Compounds with n = 2, 4, 5. SM-7
Table S4. Indicator variables and their coefficients (for Eq S3). Compounds with n = 3, 6. SM-7
Figure S4. Plot of MOR affinities found for ureas versus those found for carbamates of the **7.1-7.12** series. SM-8
Table S5. Structures and activity data for compounds **8.10-8.84**. SM-9
Table S6. Structures and activity data for compounds **8.86-8.147**. SM-13
Table S7. Crystal structures of the μ-opioid receptor. SM-23
Table S8. Crystal structures of selected GPCRs relevant to the scope of the review. SM-23
Table S9. Crystal structures of the σ-receptors. SM-26

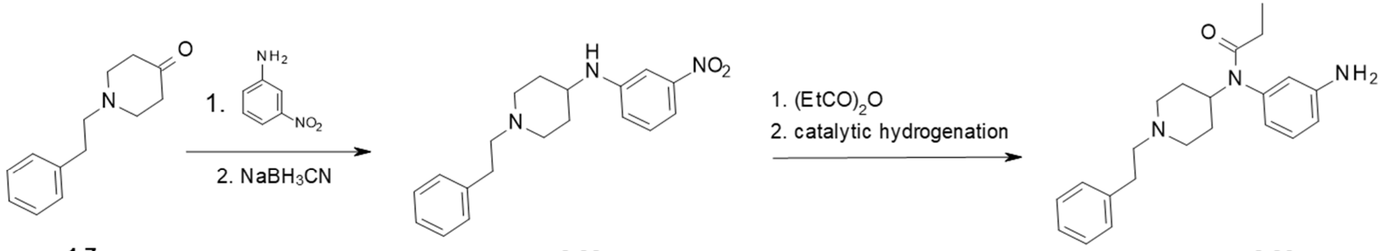

1.7
NPP

2.22

2.23

And further as in Scheme 2
of the main text.

Scheme S1. Initial synthetic steps towards compounds **2.6** and **2.7**.

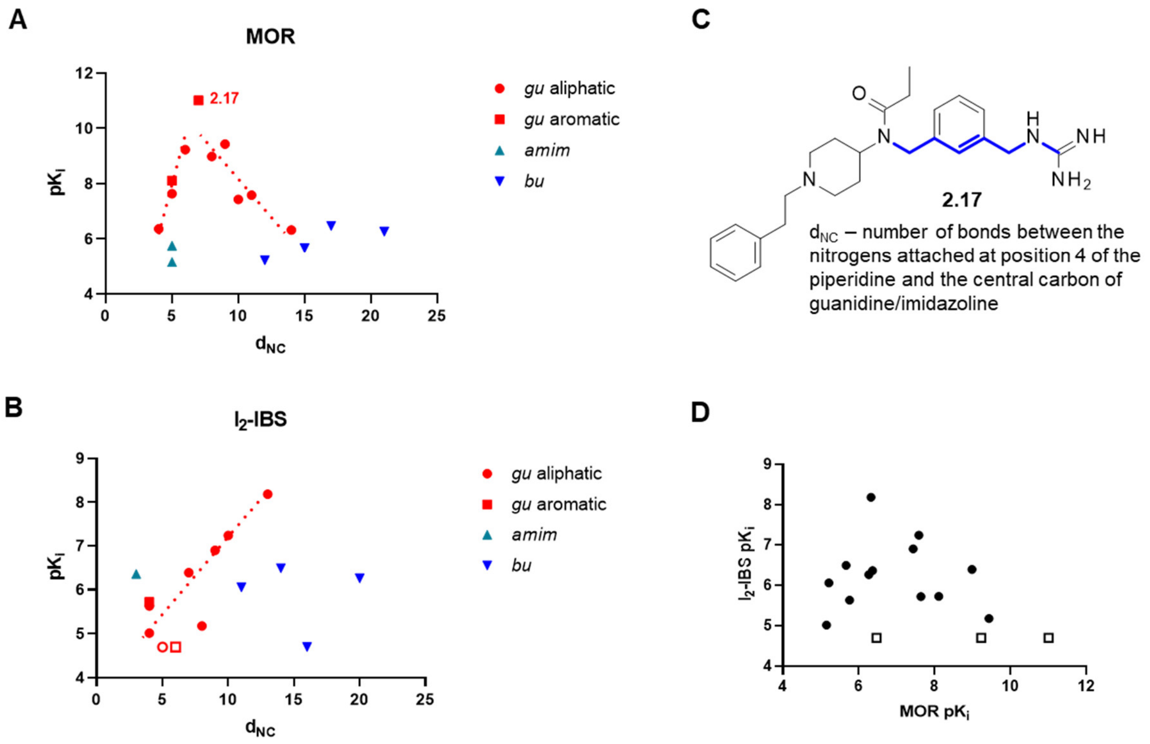

Figure S1. Correlational analysis of affinity data regarding fentanyl-related MOR/I2-IBS ligands. A) MOR: Plot of the negative logarithm of the inhibition constant (pK_i) against the linker length expressed as d_{NC}; the dashed lines mark a putative correlation. B) I_2-IBS: Plot of the negative logarithm of the inhibition constant (pK_i) against the linker length expressed as d_{NC}; the dashed lines mark a putative correlation. C) Definition of the d_{NC} variable with the example based on compound **2.17**, blue bonds are those counted for the calculation of d_{NC}, D) Plot of MOR pKi against I_2-IBS pKI. Empty points are given for compounds for which an arbitrary value of K_i = 20 μM was assigned for the purposes of the plot (reported as K_i > 10 μM).

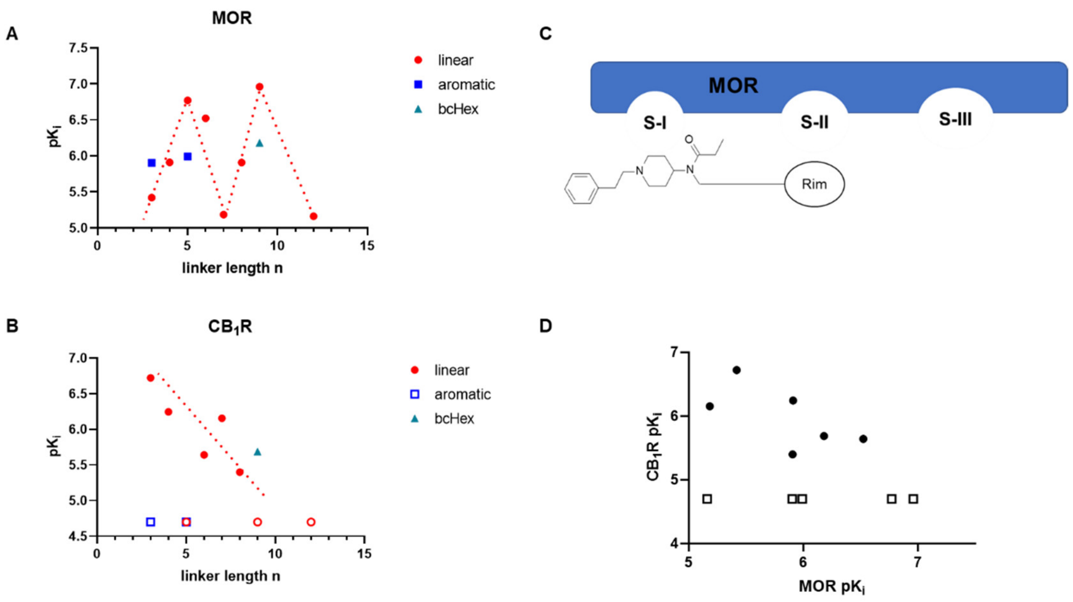

Figure S2. Correlational analysis of affinity data regarding fentanyl-related MOR/CB$_1$R ligands. A) MOR: Plot of the negative logarithm of the inhibition constant (pK$_i$) against the linker length, expressed as the number (n) of methylene units; the dashed lines mark a putative correlation. B) CB$_1$R: Plot of the negative logarithm of the inhibition constant (pK$_i$) against the linker length, expressed as the number (n) of methylene units. Empty points are given for compounds for which an arbitrary value of K$_i$ = 20 μM was assigned for the purposes of the plot (reported as K$_i$ > 10 μM). C) Hypothetical model of interactions with MOR (discussed in text). S-I is a classical set of interactions for opioid ligands, S-II and S-III represent some interaction subsites in which rimonabant fragment (Rim) could have relatively favourable contacts with MOR, D) Plot of MOR pKi against CB$_1$R pK$_I$. Empty points are given for compounds for which an arbitrary value of K$_i$ = 20 μM was assigned for the purposes of the plot (reported as K$_i$ > 10 μM).

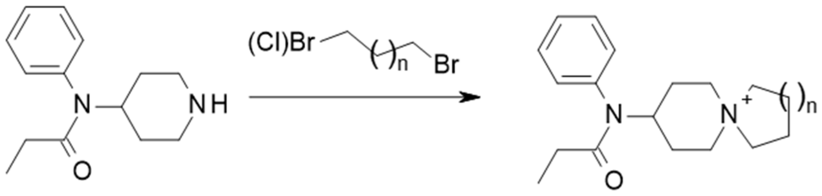

5.19

Scheme S2. Reaction of norfentanyl (**5.19**) with α,ω-bromochloroalkanes.

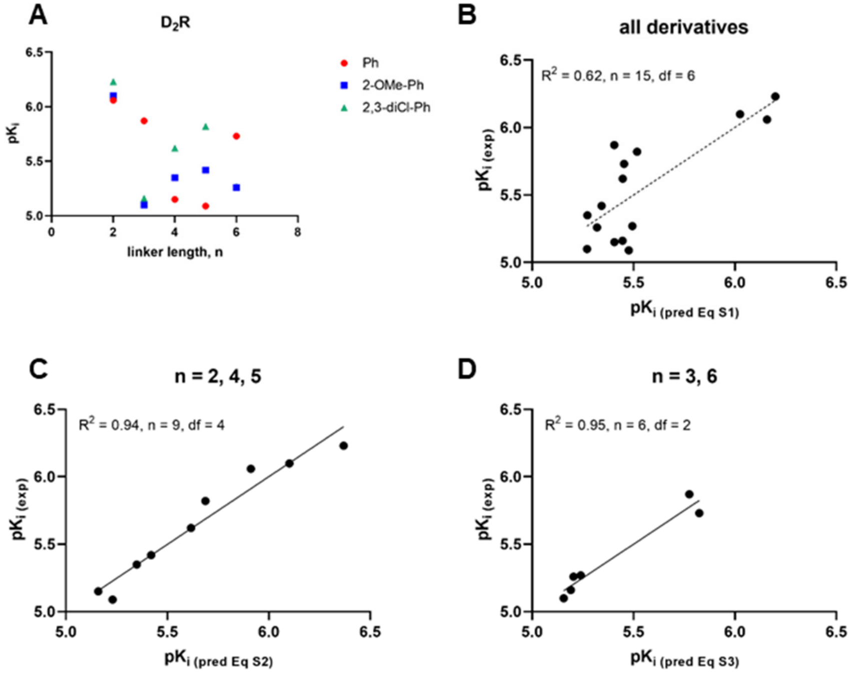

Figure S3. Correlational analysis of affinity data regarding fentanyl-related MOR/D_2-likeR ligands. A) Plot of the negative logarithm of the inhibition constant (pK_i) of D2R affinity versus the linker length (n = number of atoms, see Figure 5 and Table 5 in the main text). B-D) Plots of the experimental versus the predicted D_2R pKi's calculated with the Equations S1, S2 and S3 (see below).

The QSAR Equations based on indicator variables for MOR/D$_2$R ligands.

The QSAR equations are linear equations with indicator variables indicating presence or lack of a particular structural feature. The coefficients were obtained by linear regression.

Table S1. Indicator variables matrix for compounds **5.3** – **5.18**.

	Ar =			n =				
**cmpd**	**Ph**	**2-OMe-Ph**	**2,3-Cl$_2$-Ph**	**2**	**3**	**4**	**5**	**6**
**5.3**	1	0	0	1	0	0	0	0
**5.4**	0	1	0	1	0	0	0	0
**5.5**	0	0	1	1	0	0	0	0
**5.10**	1	0	0	0	0	1	0	0
**5.11**	0	1	0	0	0	1	0	0
**5.12**	0	0	1	0	0	1	0	0
**5.13**	1	0	0	0	0	0	1	0
**5.14**	0	1	0	0	0	0	1	0
**5.15**	0	0	1	0	0	0	1	0
**5.6**	1	0	0	0	1	0	0	0
**5.7**	0	1	0	0	1	0	0	0
**5.9**	0	0	1	0	1	0	0	0
**5.16**	1	0	0	0	0	0	0	1
**5.17**	0	1	0	0	0	0	0	1
**5.18**	0	0	1	0	0	0	0	1

Table S2. Indicator variables and their coefficients (for Eq S1).

	Ar =		n =			
**intercept**	**2-OMe-Ph**	**2,3-Cl$_2$-Ph**	**2**	**4**	**5**	**6**
-5.41	0.13	-0.04	-0.75	0.00	-0.07	-0.05
0.22	0.20	0.20	0.26	0.26	0.26	0.26

Table S3. Indicator variables and their coefficients (for Eq S2). Compounds with n = 2, 4, 5.

	Ar =		n =	
**intercept**	**2-OMe-Ph**	**2,3-Cl_2-Ph**	**2**	**4**
-5.23	-0.19	-0.46	-0.68	0.07
0.12	0.13	0.13	0.13	0.13

Table S4. Indicator variables and their coefficients (for Eq S3). Compounds with n =3, 6.

	Ar =		n =
**intercept**	**Ph**	**2,3-Cl_2-Ph**	**3**
-5.20	-0.62	-0.04	0.05
0.09	0.11	0.11	0.09

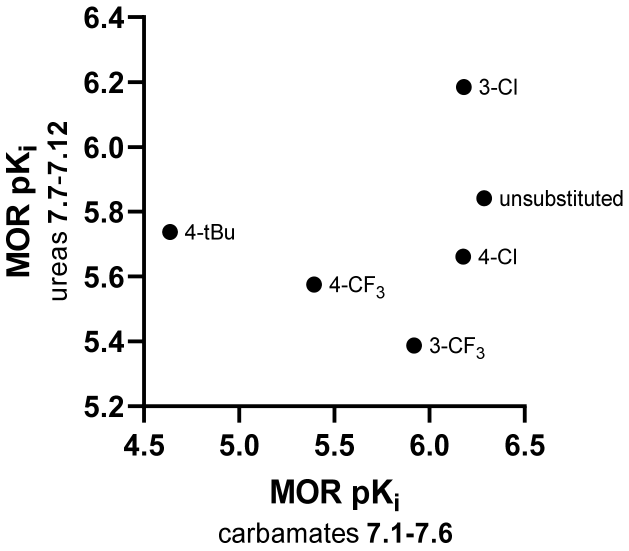

Figure S4. Plot of MOR affinities found for ureas versus those found for carbamates of the **7.1**-**7.12** series. The affinities are given as pKi (negative logarithm).

Table S5. Structures and activity data for compounds **8.10-8.84**.

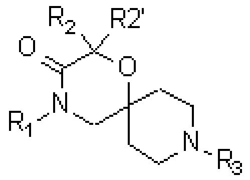

Cmpd	R_1	R_2	$R_{2'}$	R_3	K_i MOR[1] [nM]	Ki σ_1R[2] [nM]	hERG IC_{50}[3] [µM]	$\alpha_{1A}R$[4] % inhibition at 1 µM	Ref.
**8.10**	Ph	H	H	Bzl[5]	534 ± 77	26 ± 4	-	-	[1]
**8.11**	Ph	H	H	PhEth[6]	437 ± 13	57 ± 2	-	-	[1]
**8.12**	Ph	Me	H	PhEth	7 ± 6	6 ± 0.4	0.4	72	[1]
**8.13**	Ph	Me	H (S)	PhEth	69 ± 33	14 ± 0.2	0.3	68	[1]
**8.14**	Ph	Me	H (R)	PhEth	8 ± 0.5	21 ± 8	1.2	75	[1]
**8.15**	Ph	Et	H	PhEth	3 ± 2	6 ± 1	0.4	75	[1]
**8.16**	Ph	iPr	H	PhEth	1 ± 0.1	10 ± 4	1.2	65	[1]
**8.17**	Ph	CH_2OMe	H	PhEth	13 ± 3	32 ± 10	1.9	63	[1]
**8.18**	Ph	CH_2OH	H	PhEth	46 ± 12	209 ± 77	1.1	55	[1]
**8.19**	Ph	CH_2OBzl	H	PhEth	40 ± 5	40 ± 8	0.1	94	[1]
**8.20**	Ph	CH_2CO_2Me	H	PhEth	92 ± 36	54 ± 17	0.2	72	[1]
**8.21**	Ph	CH_2CO_2H	H	PhEth	>1000	>1000	-	-	[1]
**8.22**	Ph	CH_2CONMe_2	H	PhEth	636 ± 172	275 ± 4	-	-	[1]
**8.23**	Ph	Me	Me	PhEth	3 ± 1	13 ± 4	0.1	92	[1]
**8.24**	Ph	spirocyclopropyl	-	PhEth	15 ± 2	9 ± 1	1.2	87	[1]
**8.25**	2-Cl-Ph	Me	H	PhEth	3 ±1	8 ± 0.4	1.0	75	[1]
**8.26**	2-F-Ph	Me	H	PhEth	8 ± 2	11 ± 4	1.2	68	[1]
**8.27**	2-OMe-Ph	Me	H	PhEth	57 ± 7	38 ± 2	-	78	[1]
**8.28**	2-OH-Ph	Me	H	PhEth	535 ± 13	51 ± 12	-	66	[1]
**8.29**	3-OMe-Ph	Me	H	PhEth	126 ± 71	15 ± 5	1.0	82	[1]
**8.30**	3-OH-Ph	Me	H	PhEth	727 ± 583	55 ± 8	-	67	[1]
**8.31**	4-OMe-Ph	Me	H	PhEth	> 1000	14 ± 0.1	-	69	[1]
**8.32**	4-OH-Ph	Me	H	PhEth	606 ± 367	122 ± 13	-	68	[1]
**8.33**	2-Pyr[7]	Me	H	PhEth	930 ± 320	13 ± 3	-	63	[1]

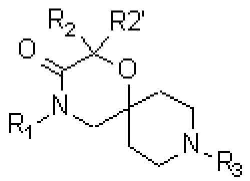

Cmpd	R_1	R_2	$R_{2'}$	R_3	K_i MOR[1]	Ki σ_1R[2]	hERG IC_{50}[3]	$\alpha_{1A}R$[4]	Ref.
					[nM]	[nM]	[µM]	% inhibition at 1 µM	
**8.34**	3-Pyr[8]	Me	H	PhEth	304 ± 76	68 ± 17	-	69	[1]
**8.35**	4-Pyr[9]	Me	H	PhEth	> 1000	88 ± 13	-	68	[1]
**8.36**	2-pyrazinyl	Me	H	PhEth	304 ± 122	13 ± 6	0.1	64	[1]
**8.37**	6-CF_3-2-Pyr	Me	H	PhEth	90 ± 36	8 ± 1	-	95	[1]
**8.38**	3-F-2-Pyr	Me	H	PhEth	93 ± 76	39 ± 6	1.2	77	[1]
**8.39**	3-CF_3-2-Pyr	Me	H	PhEth	262 ± 47	54 ± 6	8.2	60	[1]
**8.40**	5-F-3-Pyr	Me	H	PhEth	204 ± 105	3 ± 0.3	1.1	85	[1]
**8.41**	6-CF_3-3-Pyr	Me	H	PhEth	> 1000	9 ± 2	-	66	[1]
**8.42**	4-CF_3-3-Pyr	Me	H	PhEth	28 ± 19	63 ± 8	3.3	58	[1]
**8.43**	2-CF_3-3-Pyr	Me	H	PhEth	123 ± 49	48 ± 6	3.8	55	[1]
**8.44**	5-CF_3-3-Pyr	Me	H	PhEth	261 ± 115	14 ± 2	0.5	87	[1]
**8.45**	Ph	Me	H	2-F-PhEth	6 ± 3	8 ± 1	0.8	81	[1]
**8.46**	Ph	Me	H	3-F-PhEth	10 ± 3	8 ± 2	0.6	74	[1]
**8.47**	Ph	Me	H	4-F-PhEth	19 ± 7	11 ± 4	0.1	80	[1]
**8.48**	Ph	Me	H	2-acetylamino-PhEth	190 ± 14	656 ± 10	-	68	[1]
**8.49**	Ph	Me	H	3-acetylamino-PhEth	144 ± 11	147 ± 74	>10	88	[1]
**8.50**	Ph	Me	H	4-acetylamino-PhEth	>1000	237 ± 47	-	60	[1]
**8.51**	Ph	Me	H	3-CN-PhEth	49 ± 14	28 ± 8	0.6	80	[1]
**8.52**	Ph	Me	H	2-PyrEth[10]	90 ± 16	53 ± 14	2.0	49	[1]
**8.53**	Ph	Me	H	3-PyrEth[11]	117 ± 120	91 ± 28	-	67	[1]
**8.54**	Ph	Me	H	4-PyrEth[12]	516 ± 143	112 ± 35	-	30	[1]
**8.55**	4-CF_3-3-Pyr	Me	H	2-PyrEth	127 ± 78	622 ± 172	-	47	[1]
**8.56**	4-CF_3-3-Pyr	Me	H (R)	2-F-PhEth	11 ± 4	61 ± 6	6.3	54	[1]
**8.57**	3-CF_3-2-Pyr	Me	H (R)	2-F-PhEth	34 ± 11	103 ± 27	6.6	50	[1]
**8.58**	Ph	Me	H (R)	3-acetylamino-PhEth	46 ± 7	205 ± 50	>10	90	[1]

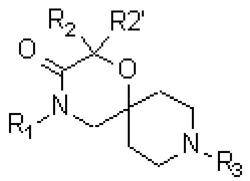

Cmpd	R_1	R_2	$R_{2'}$	R_3	K_i MOR[1]	Ki σ_1R[2]	hERG IC_{50}[3]	α_{1A}R[4]	Ref.
					[nM]	[nM]	[µM]	% inhibition at 1 µM	
**8.59**	2-CF_3-3-Pyr	spirocyclopropyl	-	2-F-PhEth	175 ± 85	58 ± 10	>10	57	[1]
**8.60**	Bzl	Me	H	PhEth	2 ± 0.3	10 ± 3	0.1	67	[2]
**8.61**	PhEth	Me	H	PhEth	20 ± 2	12 ± 2	0.2	58	[2]
**8.62**	cPr (R)	Me	H	PhEth	6 ± 1	58 ± 15	4.5	51	[2]
**8.63**	iPr	Me	H	PhEth	33 ± 5	30 ± 5	3.9	35	[2]
**8.64**	Et	Me	H	PhEth	41 ± 12	43 ± 5	3.1	27	[2]
**8.65**	Me	Me	H	PhEth	156 ± 5	142 ± 28	>10	16	[2]
**8.66**	H	Me	H	2-F-PhEth	>1000	892 ± 196	>10	7	[2]
**8.67**	Me	Me	H (R)	PhEth	91 ± 10	277 ± 76	>10	19	[2]
**8.68**	Me	Me	H (S)	PhEth	>1000	165 ± 55	-	6	[2]
**8.69**	Me	H	H	PhEth	>1000	282 ± 48	-	36	[2]
**8.70**	Me	Et	H (R)	PhEth	192 ± 41	139 ± 16	6.6	13	[2]
**8.71**	Me	iPr	H	PhEth	115 ± 39	122 ± 24	6.2	29	[2]
**8.72**	Me	Ph	H	PhEth	>1000	242 ± 13	-	26	[2]
**8.73**	Me	Me	Me	PhEth	>1000	50 ± 5	-	37	[2]
**8.74**	Me	spirocyclopropyl	-	PhEth	221 ± 51	161 ± 43	3.6	41	[2]
**8.75**	Et	Me	H (R)	PhEth	26 ± 4	66 ± 19	4.7	14	[2]
**8.76**	Et	Me	H (R)	2-F-PhEth	18 ± 7	68 ± 18	4.0	15	[2]
**8.77**	Et	Me	H (R)	3-F-PhEth	56 ± 17	64 ± 30	6.6	42	[2]
**8.78**	Et	Me	H (R)	4-F-PhEth	164 ± 29	76 ± 12	1.5	21	[2]
**8.79**	Et	Me	H (R)	2-F-3-F-PhEth	22 ± 10	556 ± 266	>10	8	[2]
**8.80**	Et	Me	H (R)	2-F-4-F-PhEth	62 ± 25	94 ± 12	2.5	11	[2]
**8.81**	Et	Me	H (R)	2-F-5-F-PhEth	64 ± 5	118 ± 7	>10	21	[2]
**8.82**	Et	Me	H (R)	2-F-6-F-PhEth	21 ± 6	609 ± 253	-	10	[2]
**8.83**	Et	spirocyclopropyl	-	2-PyrEth	>1000	783 ± 300	-	0	[2]

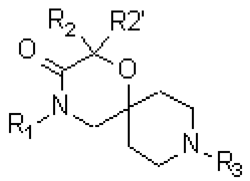

**Cmpd**	**R$_1$**	**R$_2$**	**R$_{2'}$**	**R$_3$**	**K$_i$ MOR[1]**	**Ki σ$_1$R[2]**	**hERG IC$_{50}$[3]**	**α$_{1A}$R[4]**	**Ref.**
					**[nM]**	**[nM]**	**[μM]**	**% inhibition at 1 μM**	
**8.84**	Et	Me	H (S)	2-F-5-F-PhEth	>1000	135 ± 22	>10	9	[2]

[1] competitive assays done in transfected CHO-K1 cell membranes, 1 nM [^{3}H]DAMGO as radioligand, [2] competitive assays done in transfected HEK293 cell membranes, [^{3}H]-(+)-pentazocine as radioligand, radioligand concentration 3 nM [1] or 5 nM [2], [3] whole-cell patch clamp hERG blockade, measured in CHO cells stably expressing hERG channels, [4] competitive assays done in human membranes enriched with α$_{1A}$ adrenoreceptor, 0.2 nM [^{3}H]prazosin as radioligand, [5] Bzl – benzyl, [6] PhEth - 2-phenethyl, [7] 2-Pyr – 2-pyridyl, [8] 3-Pyr – 3-pyridyl, [9] 4-Pyr – 4-pyridyl, [10] 2-PyrEth – 2-(2-pyridyl)ethyl, [11] 3-PyrEth – 2-(3-pyridyl)ethyl, [12] 4-PyrEth – 2-(4-pyridyl)ethyl.

Table S6. Structures and activity data for compounds **8.86-8.147**.

	Structure 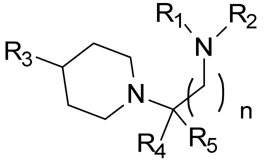						Affinity [K_i (nM)]		
**Cmpd**	**R_1**	**R_2**	**n**	**R_3**	**R_4**	**R_5**	**σ_1R[1]**	**MOR[2]**	**Reference**
**8.86**	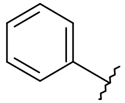	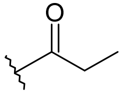	1	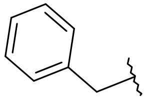	H	H	38.6 ± 1.7	3.7 ± 0.23	[3]
**8.87**	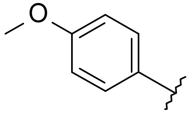	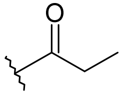	1	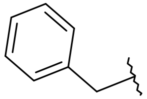	H	H	1.5 ± 0.07	20.4 ± 1.1	[3]
**8.88**	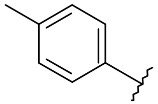	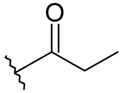	1	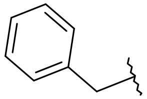	H	H	1.6 ± 0.22	16.7 ± 0.94	[3]
**8.89**	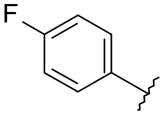	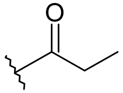	1	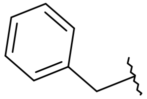	H	H	4.1 ± 0.35	19.4 ± 0.72	[3]
**8.90**	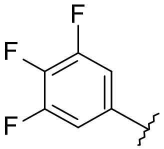	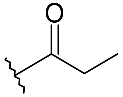	1	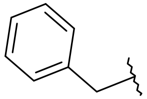	H	H	573 ± 37	> 2000	[3]
**8.91**	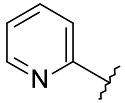	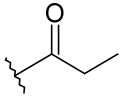	1	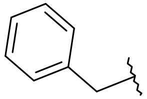	H	H	402 ± 34	238 ± 28	[3]

	Structure 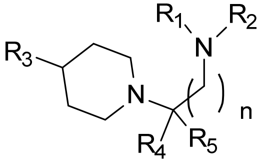						Affinity [K_i (nM)]		
**Cmpd**	**R_1**	**R_2**	**n**	**R_3**	**R_4**	**R_5**	**σ_1R[1]**	**MOR[2]**	**Reference**
**8.92**	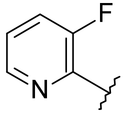	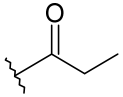	1	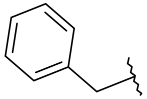	H	H	506 ± 28	253 ± 12	[3]
**8.93**	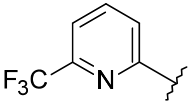	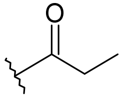	1	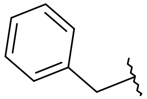	H	H	835 ± 132	> 2000	[3]
**8.94**	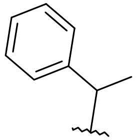	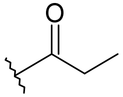	1	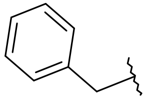	H	H	17.4 ± 1.9	694 ± 49	[3]
**8.95**	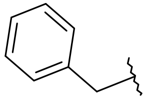	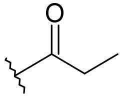	1	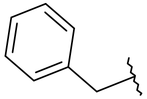	H	H	18.7 ± 0.9	573 ± 89	[3]
**8.96**	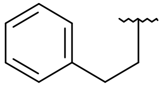	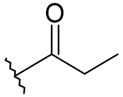	1	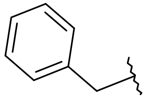	H	H	269 ± 18	> 2000	[3]
**8.97**	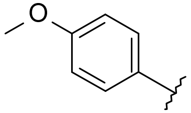	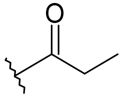	2	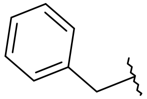	H	H	2.2 ± 0.16	43 ± 3.4	[3]
**8.98**	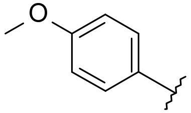	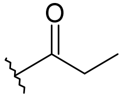	3	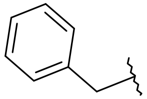	H	H	18 ± 1.92	78 ± 6.3	[3]

	Structure						Affinity [K_i (nM)]		
	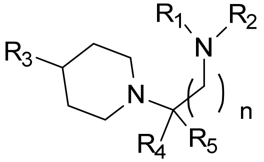								
**Cmpd**	**R_1**	**R_2**	**n**	**R_3**	**R_4**	**R_5**	**σ_1R^1**	**MOR^2**	**Reference**
**8.99**	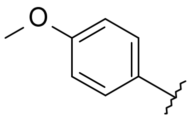	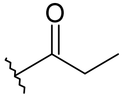	4	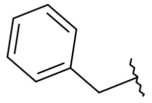	H	H	42 ± 4.8	165 ± 2.4	[3]
**8.100**	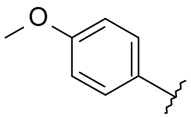	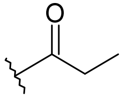	1	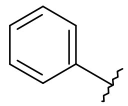	H	H	39 ± 4.7	26 ± 1.7	[3]
**8.101**	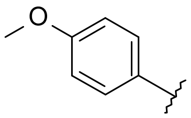	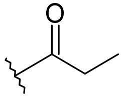	1	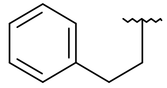	H	H	372 ± 32	82 ± 9.3	[3]
**8.102**	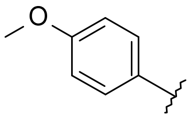	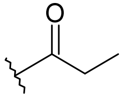	1	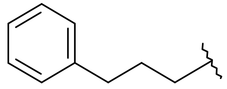	H	H	893 ± 74	74 ± 3.1	[3]
**8.103**	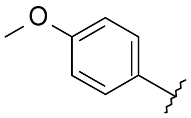	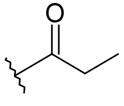	1	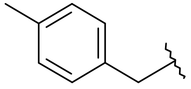	H	H	35 ± 1.6	77 ± 7.4	[3]
**8.104**	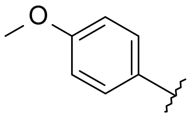	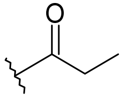	1	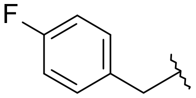	H	H	1.86 ± 0.05	2.1 ± 0.16	[3]
**8.105**	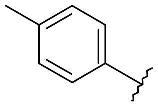	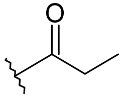	1	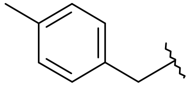	H	H	29 ± 1.3	82 ± 19	[3]

	Structure						Affinity [K_i (nM)]		
	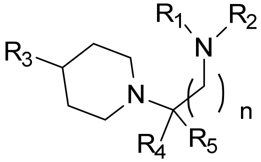								
**Cmpd**	**R_1**	**R_2**	**n**	**R_3**	**R_4**	**R_5**	**σ_1R^1**	**MOR^2**	**Reference**
**8.106**	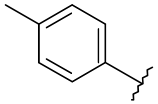	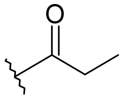	1	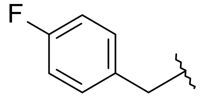	H	H	2.4 ± 0.32	27.9 ± 3.8	[3]
**8.107**	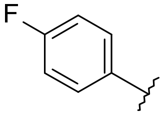	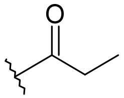	1	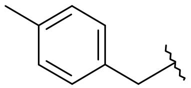	H	H	47 ± 2.4	148 ± 26	[3]
**8.108**	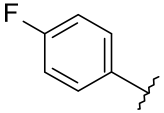	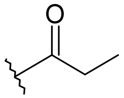	1	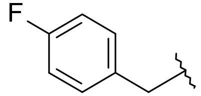	H	H	1.3 ± 0.22	5.6 ± 0.36	[3]
**8.109**	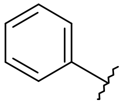	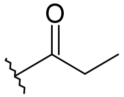	1	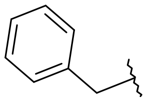	Me	Me	155.9 ± 10.3	> 1000	[4]
**8.110**	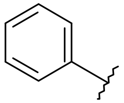	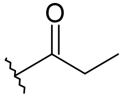	1	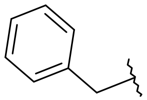	H	Me	16.3 ± 2.7	9.4 ± 0.75	[4]
**8.111**	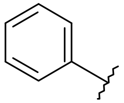	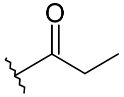	1	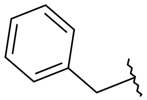	H	Et	112.4 ± 6.5	20.7 ± 2.9	[4]
**8.112**	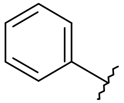	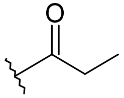	1	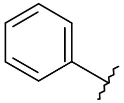	H	Me	122.9 ± 4.6	0.22 ± 0.03	[4]

	Structure 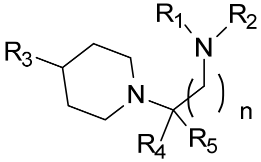						Affinity [K_i (nM)]		
**Cmpd**	**R_1**	**R_2**	**n**	**R_3**	**R_4**	**R_5**	**σ_1R[1]**	**MOR[2]**	**Reference**
**8.113**	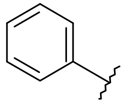	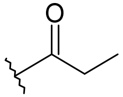	1	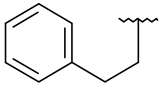	H	Me	41.5 ± 1.2	26.5 ± 2.6	[4]
**8.114**	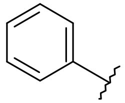	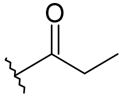	1	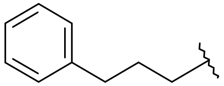	H	Me	59.8 ± 4.8	960.1 ± 15.4	[4]
**8.115**	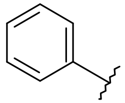	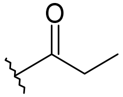	1	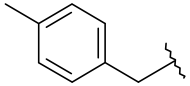	H	Me	168.1 ± 12.9	90.3 ± 3.2	[4]
**8.116**	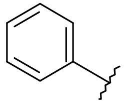	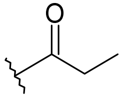	1	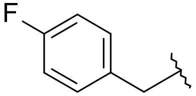	H	Me	178.0 ± 11.5	33.6 ± 2.7	[4]
**8.117**	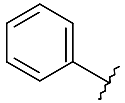	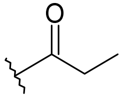	1	H	H	Me	> 1000	189.7 ± 12.5	[4]
**8.118**	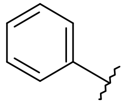	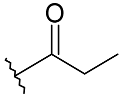	1	Me	H	Me	>1000	>1000	[4]
**8.119**	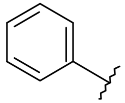	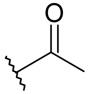	1	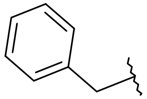	H	Me	113.4 ± 10.1	906.3 ± 43.2	[4]

	Structure						Affinity [K_i (nM)]		
	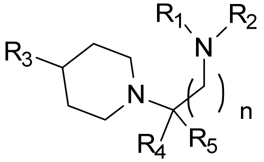								
**Cmpd**	**R_1**	**R_2**	**n**	**R_3**	**R_4**	**R_5**	**σ_1R[1]**	**MOR[2]**	**Reference**
**8.120**	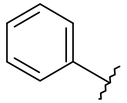	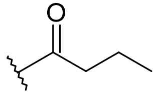	1	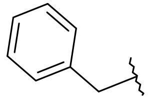	H	Me	572.4 ± 14.7	90.2 ± 5.4	[4]
**8.121**	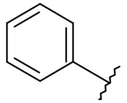	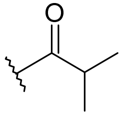	1	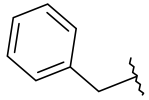	H	Me	262.8 ± 15.9	359.2 ± 25.8	[4]
**8.122**	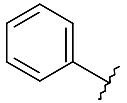	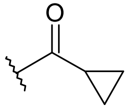	1	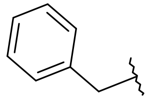	H	Me	NT	> 1000	[4]
**8.123**	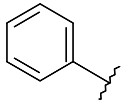	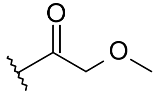	1	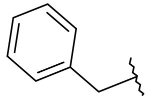	H	Me	186.7 ± 10.2	5.0 ± 0.82	[4]
**8.124**	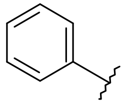	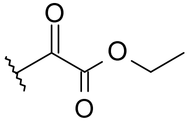	1	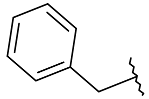	H	Me	NT	> 1000	[4]
**8.125**	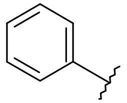	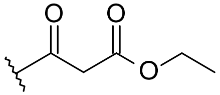	1	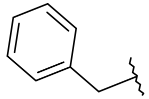	H	Me	NT	> 1000	[4]
**8.126**	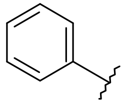	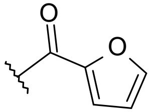	1	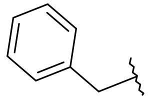	H	Me	34.9 ± 1.7	3.7 ± 1.0	[4]

	Structure						Affinity [Ki (nM)]		
	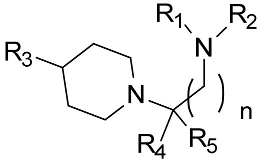								
**Cmpd**	**R_1**	**R_2**	**n**	**R_3**	**R_4**	**R_5**	**$\sigma_1 R$**[1]	**MOR**[2]	**Reference**
**8.127**	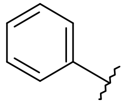	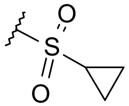	1	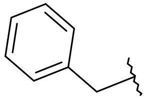	H	Me	NT	> 1000	[4]
**8.128**	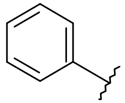	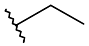	1	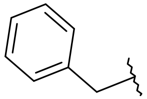	H	Me	NT	608.2 ± 4.2	[4]
**8.129**	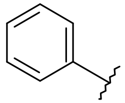	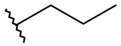	1	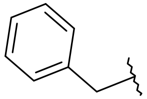	H	Me	NT	> 1000	[4]
**8.130**	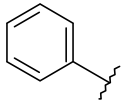	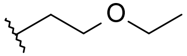	1	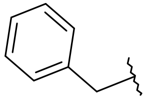	H	Me	NT	> 1000	[4]
**8.131**	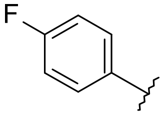	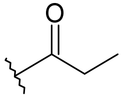	1	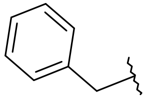	H	Me	5.5 ± 0.18	4.4 ± 0.15	[4]
**8.132**	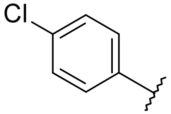	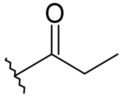	1	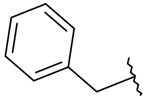	H	Me	67.1 ± 3.6	3.3 ± 0.74	[4]
**8.133**	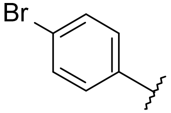	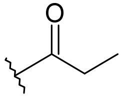	1	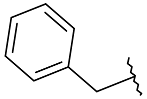	H	Me	79.7 ± 5.4	116.0 ± 6.8	[4]

	Structure<br>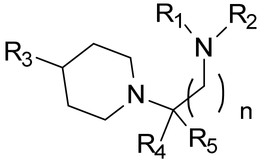						Affinity [K_i (nM)]		
**Cmpd**	**R_1**	**R_2**	**n**	**R_3**	**R_4**	**R_5**	**σ_1R[1]**	**MOR[2]**	**Reference**
**8.134**	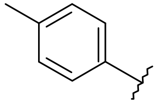	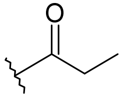	1	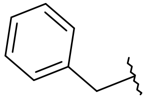	H	Me	218.6 ± 11.5	6.1 ± 0.93	[4]
**8.135**	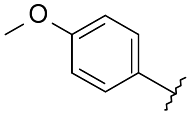	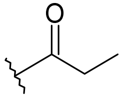	1	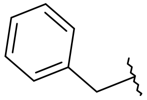	H	Me	222.0 ± 18.3	34.3 ± 1.3	[4]
**8.136**	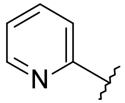	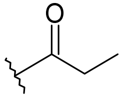	1	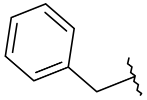	H	Me	272.0 ± 18.2	739.1 ± 25.9	[4]
**8.137**	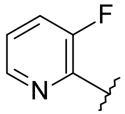	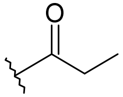	1	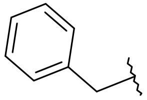	H	Me	NT	> 1000	[4]
**8.138**	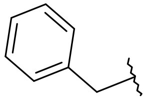	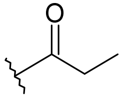	1	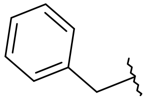	H	Me	30.2 ± 2.6	968.8 ± 39.5	[4]
**8.139**	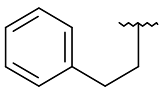	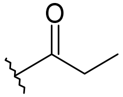	1	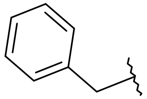	H	Me	NT	> 1000	[4]
**8.140**	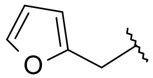	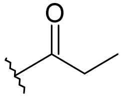	1	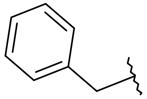	H	Me	409.5 ± 6.8	1.1 ± 0.03	[4]

	Structure<br>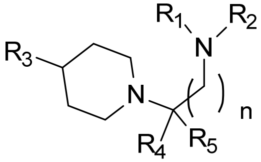						Affinity<br>[K_i (nM)]		
**Cmpd**	**R_1**	**R_2**	**n**	**R_3**	**R_4**	**R_5**	**σ_1R^1**	**MOR^2**	**Reference**
**8.141**	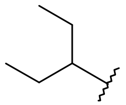	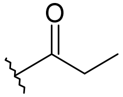	1	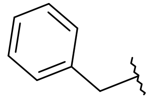	H	Me	NT	> 1000	[4]
**8.142**	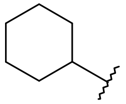	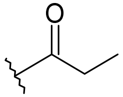	1	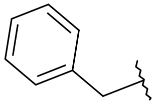	H	Me	NT	> 1000	[4]
**8.143**	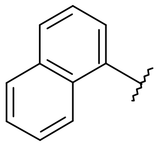	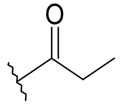	1	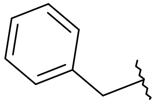	H	Me	22.8 ± 1.7	71.0 ± 3.0	[4]
**8.144**	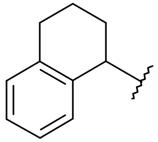	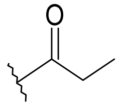	1	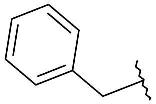	H	Me	NT	> 1000	[4]
**8.145**	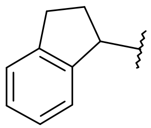	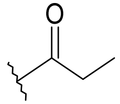	1	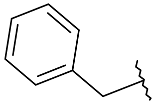	H	Me	191.2 ± 14.3	861.7 ± 16.4	[4]
**8.146**	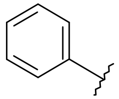	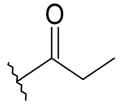	1	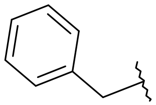	H	Me<br>(*S*)-configuration	19.4 ± 0.78	4.3 ± 0.15	[4]

	Structure<br>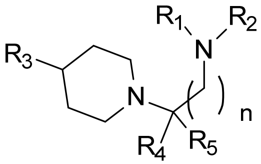						Affinity<br>[K_i (nM)]		
**Cmpd**	**R_1**	**R_2**	**n**	**R_3**	**R_4**	**R_5**	**σ_1R[1]**	**MOR[2]**	**Reference**
**8.147**	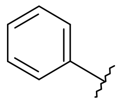	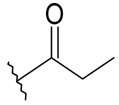	1	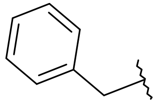	H	Me<br>(*R*)-configuration	24.2 ± 1.6	893.2 ± 12.9	[4]

[1] competitive assays done in membrane preparation from guinea pig brain, [^{3}H]-(+)-pentazocine as radioligand, [2] competitive assays done in membranes from CHO cells expressing MOR and [^{3}H]diprenorphine as radioligand (in Ref. [3]) or competitive assays done in membranes from rat brains and [^{3}H]DAMGO as radioligand (in Ref. [4]).

Table S7. Crystal structures of the μ-opioid receptor.

		STRUCTURE				LIGAND			
**IUPHAR**	**Receptor family**	**Method**	**PDB**	**Resolution**	**State**	**Name**	**Type**	**Function**	**Reference**
μ	Opioid	X-ray	4DKL	2.8	Inactive	β-FNA	small molecule	Antagonist	[5]
μ	Opioid	X-ray	5C1M	2.1	Active	BU72	small molecule	Agonist	[6]
μ	Opioid	cryo-EM	6DDE	3.5	Active	DAMGO	peptide	Agonist	[7]
μ	Opioid	cryo-EM	6DDF	3.5	Active	DAMGO	peptide	Agonist	[7]

Table S8. Crystal structures of selected GPCRs relevant to the scope of the review.

		STRUCTURE				LIGAND			
**IUPHAR**	**Receptor family**	**Method**	**PDB**	**Resolution**	**State**	**Name**	**Type**	**Function**	**Reference**
NK1	Tachykinin	X-ray	6E59	3.4	Inactive	L760735	small molecule	Antagonist	[8]
NK1	Tachykinin	X-ray	6HLL	3.3	Inactive	CP-99,994	small molecule	Antagonist	[9]
NK1	Tachykinin	X-ray	6HLO	2.4	Inactive	Aprepitant	small molecule	Antagonist	[9]
NK1	Tachykinin	X-ray	6HLP	2.2	Inactive	Netupitant	small molecule	Antagonist	[9]
NK1	Tachykinin	X-ray	6J20	2.7	Inactive	Aprepitant	small molecule	Antagonist	[10]
NK1	Tachykinin	X-ray	6J21	3.2	Inactive	Aprepitant	small molecule	Antagonist	[10]
NK1	Tachykinin	cryo-EM	7P00	2.7	Active	Substance P	peptide	Agonist	[11]

		STRUCTURE				LIGAND			
IUPHAR	Receptor family	Method	PDB	Resolution	State	Name	Type	Function	Reference
NK1	Tachykinin	cryo-EM	7P02	2.9	Active	Substance P	peptide	Agonist	[11]
NK1	Tachykinin	cryo-EM	7RMG	3	Active	Substance P	peptide	Agonist	[12]
NK1	Tachykinin	cryo-EM	7RMH	3.1	Active	Substance P	peptide	Agonist	[12]
NK1	Tachykinin	cryo-EM	7RMI	3.2	Active	Substance P 6-11	peptide	Agonist	[12]
D4	Dopamine	X-ray	5WIU	2	Inactive	Nemonapride	small molecule	Antagonist	[13]
D4	Dopamine	X-ray	5WIV	2.1	Inactive	Nemonapride	small molecule	Antagonist	[13]
D4	Dopamine	X-ray	6IQL	3.5	Inactive	L745870	small molecule	Antagonist	[14]
D3	Dopamine	X-ray	3PBL	2.9	Inactive	Eticlopride	small molecule	Antagonist	[15]
D3	Dopamine	cryo-EM	7CMU	3	Active	Pramipexole	small molecule	Agonist	[16]
D3	Dopamine	cryo-EM	7CMV	2.7	Active	PD128907	small molecule	Agonist	[16]
D2	Dopamine	X-ray	6CM4	2.9	Inactive	Risperidone	small molecule	Inverse agonist	[17]
D2	Dopamine	X-ray	6LUQ	3.1	Inactive	Haloperidol	small molecule	Antagonist	[18]
D2	Dopamine	cryo-EM	6VMS	3.8	Active	Bromocriptine	small molecule	Agonist	[19]
D2	Dopamine	X-ray	7DFP	3.1	Inactive	Spiperone	small molecule	Antagonist	[20]
D2	Dopamine	cryo-EM	7JVR	2.8	Active	Bromocriptine	small molecule	Agonist	[21]

		STRUCTURE				LIGAND			
**IUPHAR**	**Receptor family**	**Method**	**PDB**	**Resolution**	**State**	**Name**	**Type**	**Function**	**Reference**
CB1	Cannabinoid	X-ray	5TGZ	2.8	Inactive	AM-6538	small molecule	Antagonist	[22]
CB1	Cannabinoid	X-ray	5U09	2.6	Inactive	Taranabant	small molecule	Inverse agonist	[23]
CB1	Cannabinoid	X-ray	5XR8	3	Active	AM841	small molecule	Agonist	[24]
CB1	Cannabinoid	X-ray	5XRA	2.8	Active	AM11542	small molecule	Agonist	[24]
CB1	Cannabinoid	cryo-EM	6KPG	3	Active	AM841	small molecule	Agonist	[25]
CB1	Cannabinoid	X-ray	6KQI	3.3	Inactive	ORG27569	small molecule	NAM	[26]
CB1	Cannabinoid	cryo-EM	6N4B	3	Active	MDMB-Fubinaca	small molecule	Agonist	[27]
CB1	Cannabinoid	X-ray	7V3Z	3.3	Active	2-[(1R,2R,5R)-5-Hydroxy-2-(3-hydroxypropyl)cyclohexyl]-5-(2-methyloctan-2-yl)phenol	small molecule	Agonist	[28]

Table S9. Crystal structures of the σ-receptors.

	STRUCTURE			LIGAND	
**Receptor**	**Method**	**PDB**	**Resolution**	**Name**	**Reference**
σ1	X-ray	5HK1	2.51	PD144418	[29]
σ1	X-ray	5HK2	3.2	4-IBP	[29]
σ1	X-ray	6DJZ	3.08	haloperidol	[30]
σ1	X-ray	6DK1	3.12	(+)-pentazocine	[30]
σ1	X-ray	6DK0	2.90	NE-100	[30]
σ2	X-ray	7M93	2.94	PB28	[31]
σ2	X-ray	7M94	2.71	Roluperidone	[31]
σ2	X-ray	7M95	2.41	Z1241145220	[31]
σ2	X-ray	7M96	2.41	Z4857158944	[31]
σ2	X-ray	7MFI	2.81	Cholesterol	[31]

References

1. García, M.; Virgili, M.; Alonso, M.; Alegret, C.; Fernández, B.; Port, A.; Pascual, R.; Monroy, X.; Vidal-Torres, A.; Serafini, M.-T.; et al. 4-Aryl-1-oxa-4,9-diazaspiro[5.5]undecane Derivatives as Dual μ-Opioid Receptor Agonists and σ 1 Receptor Antagonists for the Treatment of Pain. *J. Med. Chem.* **2020**, *63*, 2434–2454.

2. García, M.; Virgili, M.; Alonso, M.; Alegret, C.; Farran, J.; Fernández, B.; Bordas, M.; Pascual, R.; Burgueño, J.; Vidal-Torres, A.; et al. Discovery of EST73502, a Dual μ-Opioid Receptor Agonist and σ 1 Receptor Antagonist Clinical Candidate for the Treatment of Pain. *J. Med. Chem.* **2020**, *63*, 15508–15526.

3. Xiong, J.; Jin, J.; Gao, L.; Hao, C.; Liu, X.; Liu, B.-F.; Chen, Y.; Zhang, G. Piperidine propionamide as a scaffold for potent sigma-1 receptor antagonists and mu opioid receptor agonists for treating neuropathic pain. *Eur. J. Med. Chem.* **2020**, *191*, 112144.

4. Xiong, J.; Zhuang, T.; Ma, Y.; Xu, J.; Ye, J.; Ma, R.; Zhang, S.; Liu, X.; Liu, B.-F.; Hao, C.; et al. Optimization of bifunctional piperidinamide derivatives as σ1R Antagonists/MOR agonists for treating neuropathic pain. *Eur. J. Med. Chem.* **2021**, *226*, 113879.

5. Manglik, A.; Kruse, A.C.; Kobilka, T.S.; Thian, F.S.; Mathiesen, J.M.; Sunahara, R.K.; Pardo, L.; Weis, W.I.; Kobilka, B.K.; Granier, S. Crystal structure of the μ-opioid receptor bound to a morphinan antagonist. *Nature* **2012**, *485*, 321–326.

6. Huang, W.; Manglik, A.; Venkatakrishnan, A.J.; Laeremans, T.; Feinberg, E.N.; Sanborn, A.L.; Kato, H.E.; Livingston, K.E.; Thorsen, T.S.; Kling,

R.C.; et al. Structural insights into μ-opioid receptor activation. *Nature* **2015**, *524*, 315–321.

7. Koehl, A.; Hu, H.; Maeda, S.; Zhang, Y.; Qu, Q.; Paggi, J.M.; Latorraca, N.R.; Hilger, D.; Dawson, R.; Matile, H.; et al. Structure of the μ-opioid receptor–Gi protein complex. *Nature* **2018**, *558*, 547–552.

8. Yin, J.; Chapman, K.; Clark, L.D.; Shao, Z.; Borek, D.; Xu, Q.; Wang, J.; Rosenbaum, D.M. Crystal structure of the human NK 1 tachykinin receptor. *Proc. Natl. Acad. Sci.* **2018**, *115*, 13264–13269.

9. Schöppe, J.; Ehrenmann, J.; Klenk, C.; Rucktooa, P.; Schütz, M.; Doré, A.S.; Plückthun, A. Crystal structures of the human neurokinin 1 receptor in complex with clinically used antagonists. *Nat. Commun.* **2019**, *10*, 17.

10. Chen, S.; Lu, M.; Liu, D.; Yang, L.; Yi, C.; Ma, L.; Zhang, H.; Liu, Q.; Frimurer, T.M.; Wang, M.-W.; et al. Human substance P receptor binding mode of the antagonist drug aprepitant by NMR and crystallography. *Nat. Commun.* **2019**, *10*, 638.

11. Thom, C.; Ehrenmann, J.; Vacca, S.; Waltenspühl, Y.; Schöppe, J.; Medalia, O.; Plückthun, A. Structures of neurokinin 1 receptor in complex with G q and G s proteins reveal substance P binding mode and unique activation features. *Sci. Adv.* **2021**, *7*.

12. Harris, J.A.; Faust, B.; Gondin, A.B.; Dämgen, M.A.; Suomivuori, C.-M.; Veldhuis, N.A.; Cheng, Y.; Dror, R.O.; Thal, D.M.; Manglik, A. Selective G protein signaling driven by substance P–neurokinin receptor dynamics. *Nat. Chem. Biol.* **2022**, *18*, 109–115.

13. Wang, S.; Wacker, D.; Levit, A.; Che, T.; Betz, R.M.; McCorvy, J.D.; Venkatakrishnan, A.J.; Huang, X.-P.; Dror, R.O.; Shoichet, B.K.; et al. D 4 dopamine receptor high-resolution structures enable the discovery of selective agonists. *Science (80-.).* **2017**, *358*, 381–386.

14. Zhou, Y.; Cao, C.; He, L.; Wang, X.; Zhang, X.C. Crystal structure of dopamine receptor D4 bound to the subtype selective ligand, L745870. *Elife* **2019**, *8*.

15. Chien, E.Y.T.; Liu, W.; Zhao, Q.; Katritch, V.; Won Han, G.; Hanson, M.A.; Shi, L.; Newman, A.H.; Javitch, J.A.; Cherezov, V.; et al. Structure of the Human Dopamine D3 Receptor in Complex with a D2/D3 Selective Antagonist. *Science (80-.).* **2010**, *330*, 1091–1095.

16. Xu, P.; Huang, S.; Mao, C.; Krumm, B.E.; Zhou, X.E.; Tan, Y.; Huang, X.-P.; Liu, Y.; Shen, D.-D.; Jiang, Y.; et al. Structures of the human dopamine D3 receptor-Gi complexes. *Mol. Cell* **2021**, *81*, 1147-1159.e4.

17. Wang, S.; Che, T.; Levit, A.; Shoichet, B.K.; Wacker, D.; Roth, B.L. Structure of the D2 dopamine receptor bound to the atypical antipsychotic drug risperidone. *Nature* **2018**, *555*, 269–273.

18. Fan, L.; Tan, L.; Chen, Z.; Qi, J.; Nie, F.; Luo, Z.; Cheng, J.; Wang, S. Haloperidol bound D2 dopamine receptor structure inspired the discovery of subtype selective ligands. *Nat. Commun.* **2020**, *11*, 1074.

19. Yin, J.; Chen, K.-Y.M.; Clark, M.J.; Hijazi, M.; Kumari, P.; Bai, X.; Sunahara, R.K.; Barth, P.; Rosenbaum, D.M. Structure of a D2 dopamine receptor–G-protein complex in a lipid membrane. *Nature* **2020**, *584*, 125–129.

20. Im, D.; Inoue, A.; Fujiwara, T.; Nakane, T.; Yamanaka, Y.; Uemura, T.; Mori, C.; Shiimura, Y.; Kimura, K.T.; Asada, H.; et al. Structure of the dopamine D2 receptor in complex with the antipsychotic drug spiperone. *Nat. Commun.* **2020**, *11*, 6442.

21. Zhuang, Y.; Xu, P.; Mao, C.; Wang, L.; Krumm, B.; Zhou, X.E.; Huang, S.; Liu, H.; Cheng, X.; Huang, X.-P.; et al. Structural insights into the human D1 and D2 dopamine receptor signaling complexes. *Cell* **2021**, *184*, 931-942.e18.

22. Hua, T.; Vemuri, K.; Pu, M.; Qu, L.; Han, G.W.; Wu, Y.; Zhao, S.; Shui, W.; Li, S.; Korde, A.; et al. Crystal Structure of the Human Cannabinoid Receptor CB1. *Cell* **2016**, *167*, 750-762.e14.

23. Shao, Z.; Yin, J.; Chapman, K.; Grzemska, M.; Clark, L.; Wang, J.; Rosenbaum, D.M. High-resolution crystal structure of the human CB1 cannabinoid receptor. *Nature* **2016**, *540*, 602–606.

24. Hua, T.; Vemuri, K.; Nikas, S.P.; Laprairie, R.B.; Wu, Y.; Qu, L.; Pu, M.; Korde, A.; Jiang, S.; Ho, J.-H.; et al. Crystal structures of agonist-bound human cannabinoid receptor CB1. *Nature* **2017**, *547*, 468–471.

25. Hua, T.; Li, X.; Wu, L.; Iliopoulos-Tsoutsouvas, C.; Wang, Y.; Wu, M.; Shen, L.; Brust, C.A.; Nikas, S.P.; Song, F.; et al. Activation and Signaling Mechanism Revealed by Cannabinoid Receptor-Gi Complex Structures. *Cell* **2020**, *180*, 655-665.e18.

26. Shao, Z.; Yan, W.; Chapman, K.; Ramesh, K.; Ferrell, A.J.; Yin, J.; Wang, X.; Xu, Q.; Rosenbaum, D.M. Structure of an allosteric modulator bound to the CB1 cannabinoid receptor. *Nat. Chem. Biol.* **2019**, *15*, 1199–1205.

27. Krishna Kumar, K.; Shalev-Benami, M.; Robertson, M.J.; Hu, H.; Banister, S.D.; Hollingsworth, S.A.; Latorraca, N.R.; Kato, H.E.; Hilger, D.; Maeda, S.; et al. Structure of a Signaling Cannabinoid Receptor 1-G Protein Complex. *Cell* **2019**, *176*, 448-458.e12.

28. Wang, X.; Liu, D.; Shen, L.; Li, F.; Li, Y.; Yang, L.; Xu, T.; Tao, H.; Yao, D.; Wu, L.; et al. A Genetically Encoded F-19 NMR Probe Reveals the Allosteric Modulation Mechanism of Cannabinoid Receptor 1. *J. Am. Chem. Soc.* **2021**, *143*, 16320–16325.

29. Schmidt, H.R.; Zheng, S.; Gurpinar, E.; Koehl, A.; Manglik, A.; Kruse, A.C. Crystal structure of the human σ1 receptor. *Nature* **2016**, *532*, 527–530.

30. Schmidt, H.R.; Betz, R.M.; Dror, R.O.; Kruse, A.C. Structural basis for σ1receptor ligand recognition. *Nat. Struct. Mol. Biol.* **2018**, *25*, 981–987.

31. Alon, A.; Lyu, J.; Braz, J.M.; Tummino, T.A.; Craik, V.; O'Meara, M.J.; Webb, C.M.; Radchenko, D.S.; Moroz, Y.S.; Huang, X.-P.; et al. Structures of the σ2 receptor enable docking for bioactive ligand discovery. *Nature* **2021**, *600*, 759–764.
